# Cellular apoptosis and necrosis as therapeutic targets for novel Eugenol Tosylate Congeners against *Candida albicans*

**DOI:** 10.1038/s41598-020-58256-4

**Published:** 2020-01-27

**Authors:** Shabir Ahmad Lone, Mohmmad Younus Wani, Pascaline Fru, Aijaz Ahmad

**Affiliations:** 1 Department of Clinical Microbiology and Infectious Diseases, School of Pathology, Faculty of Health Sciences, University of the Witwatersrand, Johannesburg, 2193 South Africa; 2 Chemistry Department, Faculty of Science, University of Jeddah, P.O. Box 80327, Jeddah, 21589 Kingdom of Saudi Arabia; 3 Department of Surgery, School of Clinical Medicine, Faculty of Health Sciences, University of the Witwatersrand, Johannesburg, 2193 South Africa; 4 Infection Control, Charlotte Maxeke Johannesburg Academic Hospital National Health Laboratory Service, Johannesburg, 2193 South Africa

**Keywords:** Drug discovery and development, Fungal pathogenesis, Infection

## Abstract

Despite the rise of new *Candida* species, *Candida albicans* tops the list with high morbidity and mortality rates. To tackle this problem there is a need to explore new antifungals that could replace or augment the current treatment options. We previously reported that tosylation of eugenol on hydroxyl group resulted in molecules with enhanced antifungal potency. In line with that work, we synthesized new eugenol tosylate congeners (ETC-1–ETC-7) with different substituents on pendent sulfonyl group and tested their susceptibility against different fluconazole susceptible and resistant *C*. *albicans* strains. We evaluated physiology and mode of cell death in response to the most active derivatives by analyzing major apoptotic markers in yeast such as phosphatidylserine externalization, DNA fragmentation, mitochondrial depolarization and decrease in cytochrome c oxidase activity. The results demonstrated that all *C*. *albicans* strains were variably susceptible to the test compounds with MIC ranging from 0.125–512 µg/ml, and the most active compounds (ETC-5, ETC-6 and ETC-7) actuate apoptosis and necrosis in *Candida* cells in a dose-dependent manner via metacaspase-dependent pathway. Furthermore haemolytic assay showed low cytotoxicity effect of these ETCs. Overall the results indicated that ETCs exhibit potential antifungal activity against *C*. *albicans* by activating apoptotic and necrotic pathways.

## Introduction

Infections with *Candida* are one of the leading global causes of deaths among sick individuals. With the advent of HIV and increase in the organ transplant and modern-day surgeries, significant increase in the incidences of invasive fungal infections caused by *Candida* species have been reported. *Candida* species ranked as 2^nd^ most common causative agents of fungal infections globally and are fifth among hospital-acquired pathogens^1,2^. Among all *Candida* species, *C*. *albicans* remains the most predominant and most pathogenic species globally^3^. With our current clinical settings, there are only few antifungal drugs available to treat the *C*. *albicans* infections. In addition, these existing antifungals are incompetent, possess narrow antifungal spectrum, have side effects and drug resistance. Therefore, there is a clear need to explore and identify new antifungal molecules with alternative and multiple mechanisms of action.

Natural products have long been approached to find new antifungals, however most of them have often been overlooked due to their adverse physicochemical characteristics. To overcome this, modification or derivatization of natural products is carried out to obtain molecules with desirable biological properties. Some modified natural compounds labelled as semi-synthetic drugs have already been reported to treat various infectious diseases^4,5^.

Eugenol has been extensively studied for its various biological actions, such as analgesic, anti-inflammatory, antioxidant, anticarcinogenic, antimutagenic, anaesthetic, antiparasitic, antibacterial, and at the top, antifungal^6^. Due to the inadequacy of antifungals and their adverse side effects, eugenol gained much attention and was broadly studied for its antifungal activities. Eugenol has also been reported to reduce adherence, biofilm formation and even alter the morphogenesis in *C*. *albicans*^7^. Eugenol has been found to significantly decrease cell viability and its fungicidal mode of action against *C*. *albicans*, could be related to its ability to disrupt cell wall integrity by increasing cell permeability, resulting in cell leakage. Furthermore, eugenol has also been found to influence ergosterol biosynthesis pathway by reducing the total intracellular sterol content and activate apoptosis in *C*. *albicans*^8^. However due to its hypersensitivity reactions and cytotoxic effects, its clinical testing has been halted. Therefore, modification of its chemical structure is sought out to be an important strategy to obtain molecules with desirable physicochemical and improved biological properties^5,9,10^. The enhanced antimicrobial activity of eugenol by its derivatisation has already been reported^11–13^. Recently, new Mannich base-type derivatives of eugenol were found to possess antifungal activity even equivalent to fluconazole^14^. In our previous study, potent antifungal activity of different eugenol tosylate derivatives and their synergistic interaction with fluconazole against different *Candida* isolates was reported^5^. In the same study it was also revealed that these eugenol derivatives inhibit ergosterol biosynthesis in *Candida* cells. Based on that study, we synthesized seven new eugenol tosylate congeners (ETCs) with different substituents on the pendent sulfonyl group and studied their antifungal activity against different *C*. *albicans* isolates. In our recent study, we reported that these novel ETCs target 14 α-demethylase (CYP51) enzyme and thereby inhibit ergosterol biosynthesis and also significantly downregulates the expression of its related gene *ERG11* in *C*. *albicans*^15^. To further understand in depth mechanisms of the antifungal action of these ETCs, apoptosis studies were done using the characteristic markers of apoptosis, such as phosphatidylserine (PS) externalization, DNA fragmentation, mitochondrial depolarization and decrease in cytochrome c oxidase activity. Apoptosis, also known as programmed cell death (PCD), is a well-controlled process in metazoans and its deregulation contributes to the pathogenesis of multiple diseases. Apoptosis can be triggered by various intrinsic and extrinsic stimuli and therefore induction of apoptosis in yeast cells is considered as powerful model for the screening of new antifungal agents and could provide basis for future therapies.

## Results

### Chemistry

**Figure 1:**
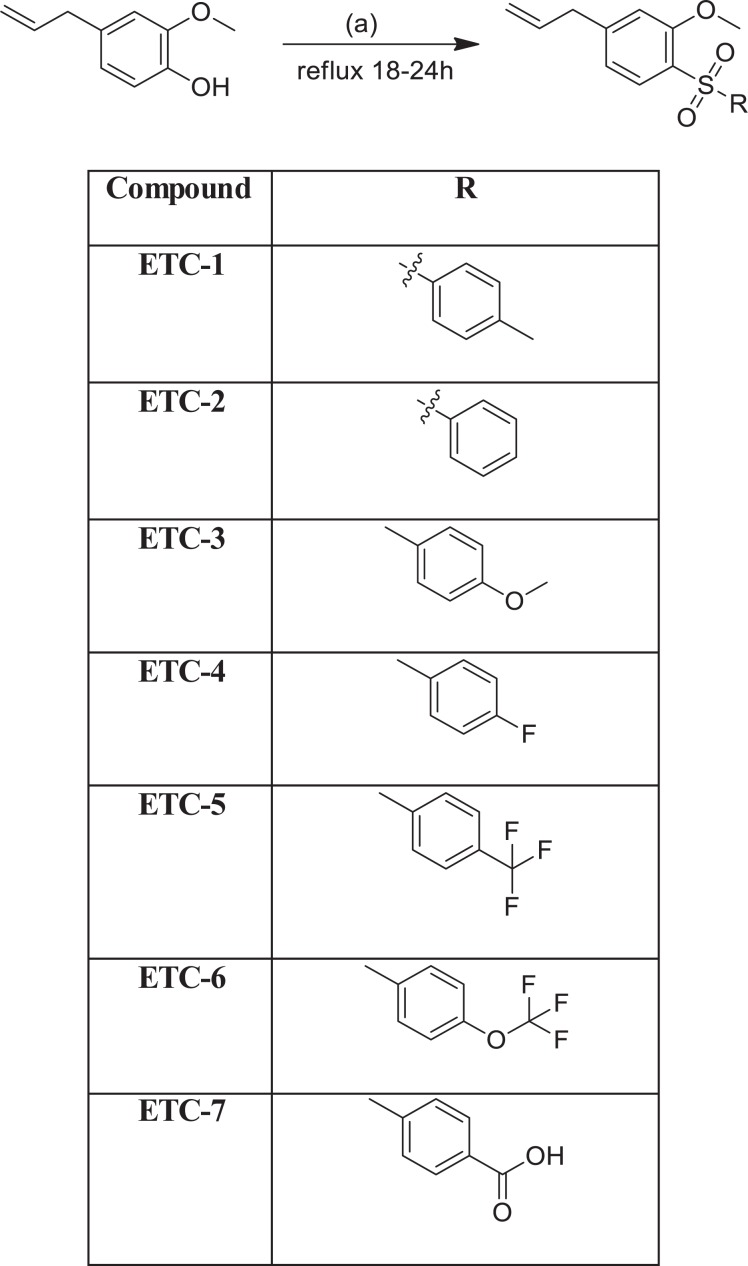
Synthesis of eugenol-tosylate congeners (ETC-1–ETC-7).

In our previous studies on eugenol, tosylation on the hydroxyl group resulted in the formation of some eugenol-tosylate derivatives with interesting antifungal properties^5^. In line with those findings, we performed tosylation of eugenol to get new tosylate derivatives, which differed on the sulfonyl functionality for structure-active relationship. The synthesis was performed following our reported procedure with some slight modifications wherever necessary^5^. Overall, seven new derivatives were obtained (Fig. 1) and structurally characterized by different physical and spectroscopic techniques. Purity of the newly synthesised compounds was confirmed by TLC and NMR (Fig. S1). Absence of any characteristic bands in the region 3200–3700 cm^−1^ in FTIR for –OH functional group and the appearance of bands around 1150–1158 cm^−1^ and 1308–1315 cm^−1^ corresponding to the sulfonyl group of the tosylates (ETC-1–ETC-7) is an ample evidence for the formation of these derivatives. Furthermore, absence of a broad peak for –OH in ^1^HNMR of all the derivatives and appearance of other characteristic peaks, also confirmed by ^13^CNMR spectra (Fig. S1). Structural confirmation of all the derivatives was done by positive ion mass spectra showing [M + H]^+^ peaks in accordance with their molecular weight (Fig. S2). The detailed data is given in experimental section.

### Physicochemical properties

In drug discovery, it is important for the test compounds to have good physiochemical properties, in order to reduce the chances of failures in the clinical trials^16,17^. These physiochemical properties play an important role in filtering out unsuitable compounds, despite their active pharmacological properties. Among the most noted physiochemical properties are pK_a_, solubility, and lipophilicity and therefore it is vital that all the test compounds fulfil these parameters for both *in silico* and *in vitro* evaluation of drug-like properties^16–19^. Knowing the importance of physiochemical properties, Lipinski formulated “Rule of Five”, according to which the efficient drug candidates should have logP ≤ 5, molecular weight ≤500, number of hydrogen bond acceptors ≤10 and number of hydrogen bond donors ≤5. All the derivatives ETC-1–ETC-7 and the parent molecule eugenol used in this study obeyed the “Rule of Five” with logP values <5 and hydrogen bond acceptor <10, hydrogen bond donor’s <5, and molecular weight <500 as shown in Table 1. Therefore, these molecules show desirable drug likeness properties which encourage their animal model studies.

**Table 1:** Lipinski’s tools for measuring drug likeness.

Compound	M.W	CLogP	LogD*	No. of HBAs^a^	No. of HBDs^a^	PSA
ETC-1	318.39	4.566	4.64	3	0	52.6
ETC-2	304.36	4.067	4.13	3	0	52.6
ETC-3	334.39	4.136	3.97	4	0	61.8
ETC-4	322.35	4.21	4.27	3	0	52.6
ETC-5	372.36	4.95	5.01	3	0	52.6
ETC-6	388.36	5.245	5.56	4	0	61.8
ETC-7	348.37	3.878	0.54	5	1	89.9
EUG	164.20	2.397	2.61	2	1	29.46

*At PH 7.4; ^a^HBA—hydrogen bond acceptor, HBD—hydrogen bond donor, PSA—polar surface area obtained by Marvin Sketch 5.1.

### Antifungal activity

The *in vitro* susceptibility of 10 clinical isolates of *C*. *albicans* along with *C*. *albicans* SC5314 against seven eugenol derivatives (ETC-1–ETC-7) were evaluated by determining MIC and MFC values. All the results are compiled in Table 2 and it was observed that all the test compounds showed improved antifungal activity when compared to their parent compound, eugenol (MIC 500 µg/ml; MFC 1000 µg/mL). The MIC and MFC values of all the test compounds (ETC-1–ETC-7) against fluconazole susceptible strains ranged from 0.125–256 µg/mL and 0.5–512 µg/mL respectively, and for fluconazole resistant strains the values ranged from 0.25–512 µg/mL and 1–1024 µg/mL respectively. The order of antifungal potency based on susceptibility results is ETC-5 > ETC-6 > ETC-7 > ETC-1 > ETC-4 > ETC-2 > ETC-3. MIC values of fluconazole against susceptible and resistant strains were 0.125–0.25 µg/mL and 8–64 µg/mL, respectively, and have therefore been categorised based on the cut off points as recommended by CLSI^20^. Among the test compounds ETC-5 (MIC = 0.125–0.5 µg/mL; MFC 0.5–2 µg/mL), ETC-6 (MIC 0.25–1 µg/mL; MFC 1–2 µg/mL) and ETC-7 (MIC 1–4 µg/mL; MFC 2–8 µg/mL) exhibited the most potent *in vitro* antifungal activity not only against fluconazole susceptible *C*. *albicans* strains but also against fluconazole resistant isolates and were therefore selected for further studies.

**Table 2:** Minimum inhibitory concentrations and minimum fungicidal concentrations (µg/ml) of eugenol and 7 newly synthesized compounds eugenol tosylate congeners (ETC-1–ETC-7) against different fluconazole susceptible and resistant strains of *Candida albicans*.

Compounds		*Candida albicans* strains										
		FLC Susceptible strains						FLC Resistant strains				
		SC5314	4175	4179	4180	4563	4576	5112	4135	4122	4324	4106
EUG	MIC	500	500	500	500	500	500	500	500	500	500	500
	MFC	1000	1000	1000	1000	1000	1000	1000	1000	1000	1000	1000
ETC-1	MIC	16	32	64	32	64	16	128	128	64	64	32
	MFC	32	64	128	64	256	32	256	256	128	256	64
ETC-2	MIC	32	32	128	64	256	128	256	128	256	128	64
	MFC	64	64	256	256	512	256	512	512	512	256	256
ETC-3	MIC	64	64	256	128	256	256	512	512	256	256	128
	MFC	128	128	512	512	512	512	1024	1024	512	512	256
ETC-4	MIC	16	32	128	64	128	64	128	128	128	64	64
	MFC	32	64	256	128	256	128	256	256	256	128	128
ETC-5	MIC	0.125	0.125	0.25	0.5	0.125	0.25	1	0.5	0.5	0.25	0.25
	MFC	0.5	0.5	1	2	0.5	1	4	2	2	1	1
ETC-6	MIC	0.25	0.25	0.5	1	0.25	0.5	2	1	1	0.5	0.5
	MFC	1	1	2	2	1	2	8	4	2	4	2
ETC-7	MIC	1	1	2	4	1	2	8	4	8	4	2
	MFC	2	2	4	8	2	4	16	8	16	8	4
FLC	MIC	0.25	0.125	0.25	0.25	0.25	0.25	64	32	32	16	8

### Apoptosis

#### Phosphatidylserine (PS) externalization

Cells after exposure to various concentrations of the active compounds (ETC-5, ETC-6, ETC-7) were analysed by double staining with annexin V-FITC and propidium iodide (PI). The apoptotic cells with externalized PS were detected by annexin V-FITC, whereas necrotic cells were detected by PI staining.

**Figure 2:**
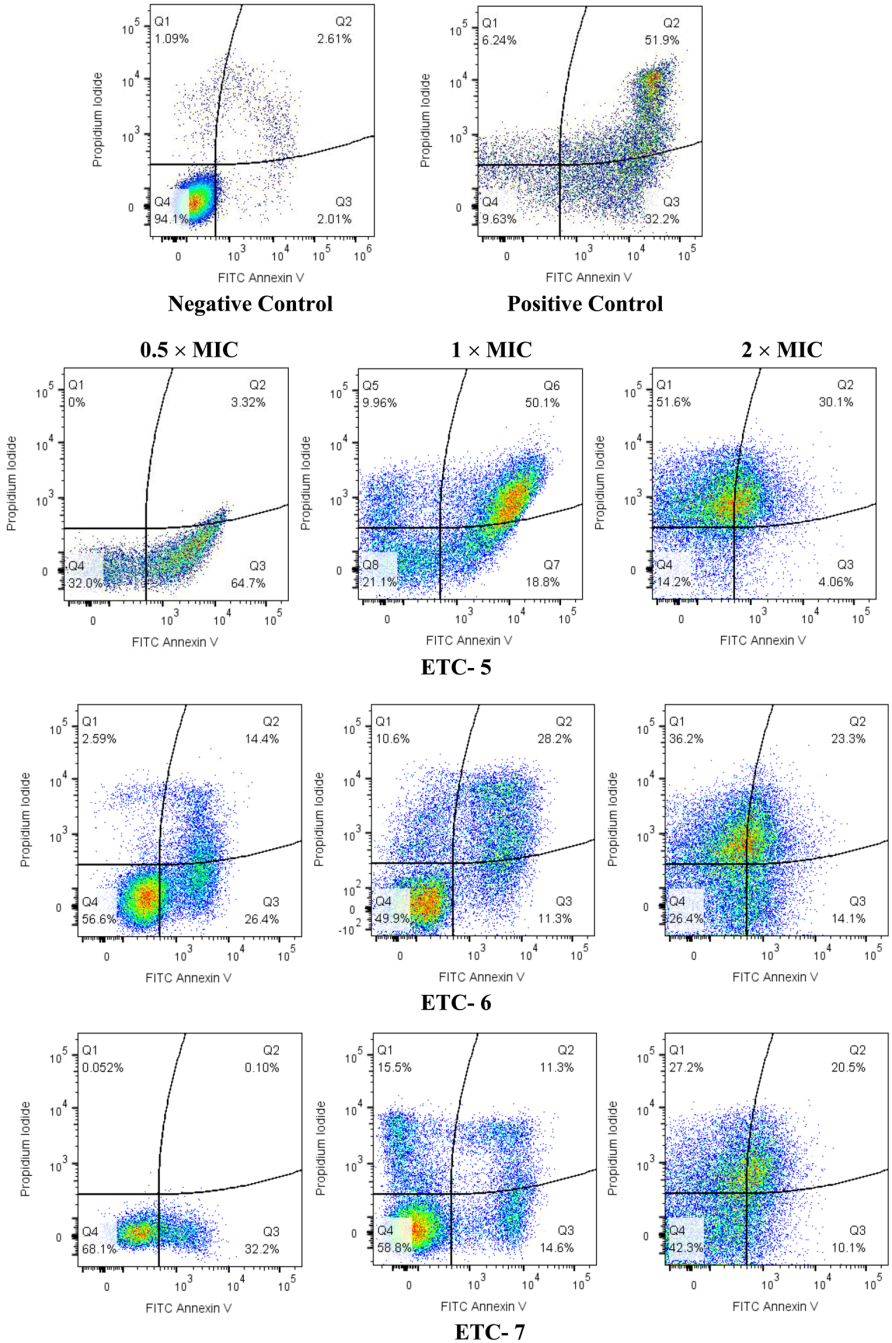
Cell apoptosis measured by flowcytometry using annexin V-FITC and PI double staining. *C*. *albicans* SC5314 cells were exposed to varying concentrations of test compounds (ETC-5, ETC-6 and ETC-7). Untreated cells and cells exposed to H_2_O_2_ (250 μmol/L) were used as negative and positive controls respectively. In each density plot quadrant Q1: shows necrotic cells (annexin− PI+); Q2: late apoptotic cells (annexin+ PI+); Q3: early apoptotic cells (annexin+ PI−) and Q4: shows viable cells (annexin− PI−).

**Figure 3:**
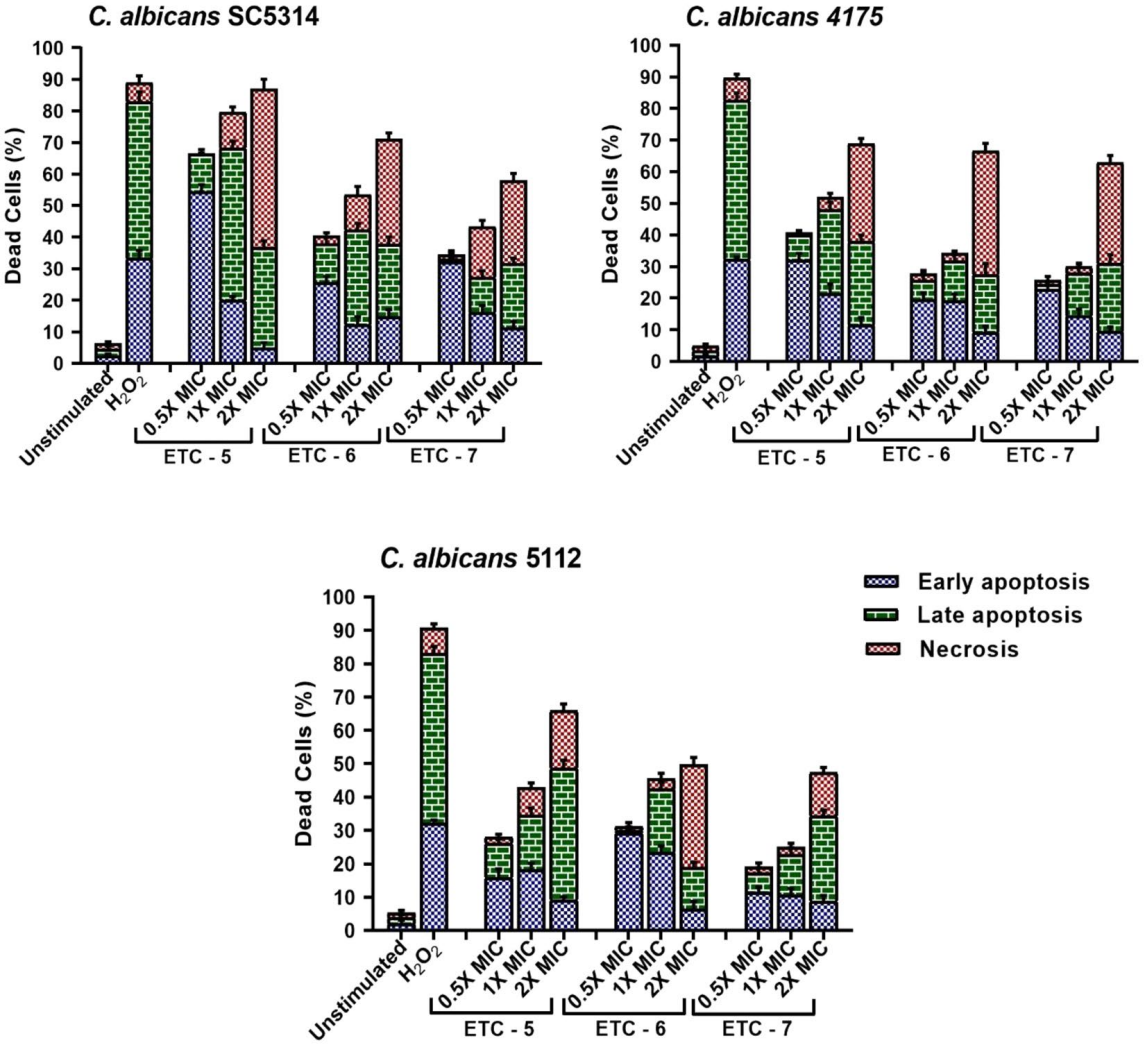
Percentage of cells showing early apoptosis, late apoptosis and necrosis after being exposed to 0.5 × MIC, 1 × MIC and 2 × MIC of ETC-5, ETC-6 and ETC-7. Untreated cells and cells exposed to H_2_O_2_ (250 μmol/L) were used as negative and positive controls, respectively. Data are presented from three independent experiments using the means ± S.D.

Three *C*. *albicans* strains including fluconazole susceptible *C*. *albicans* 4175, fluconazole resistant *C*. *albicans* 5112 and a control strain *C*. *albicans* SC5314 were exposed to 0.5 × MIC, 1 × MIC and 2 × MIC of ETC-5, ETC-6 and ETC-7. Representative dot plots of *C*. *albicans* SC5314 treated with three different concentrations of the active compounds along with positive and negative controls are presented in Fig. 2. Dot plots representing *C*. *albicans* 4175 and *C*. *albicans* 5112 treated with three different concentrations of the active compounds are presented in Fig. S3. Among the test compounds, ETC-5 was the most active compound against all the strains of *C*. *albicans* to induce apoptosis followed by ETC-6 and ETC-7. ETC-5 showed early apoptosis (5–54%), late apoptosis (8–48%) and necrosis (2–51%) (Fig. 3). These figures for ETC-6 are; early apoptosis (6–29%), late apoptosis (2–30%) and necrosis (2–39%) and for ETC-7 are; early apoptosis (8–32%), late apoptosis (2–26%) and necrosis (1–31%) (Fig. 3). The positive control (250 μmol/L H_2_O_2_) showed early apoptosis (32–33%), late apoptosis (50–51%) and necrosis (6–7%), whereas negative control (unstimulated cells) resulted in early apoptosis (1–2%), late apoptosis (1–2%) and necrosis (1–2%) (Fig. 3). The results also show that cell death was dose-dependent with a significant increase in the percentage of early apoptosis, late apoptosis and in necrosis compared to unstimulated control. Therefore, these results indicated that the most active derivatives (ETC-5, ETC-6 and ETC-7) induce both apoptosis and necrosis by phosphatidylserine externalization and membrane disruption in a dose-dependent manner.

#### DNA damage

**Figure 4:**
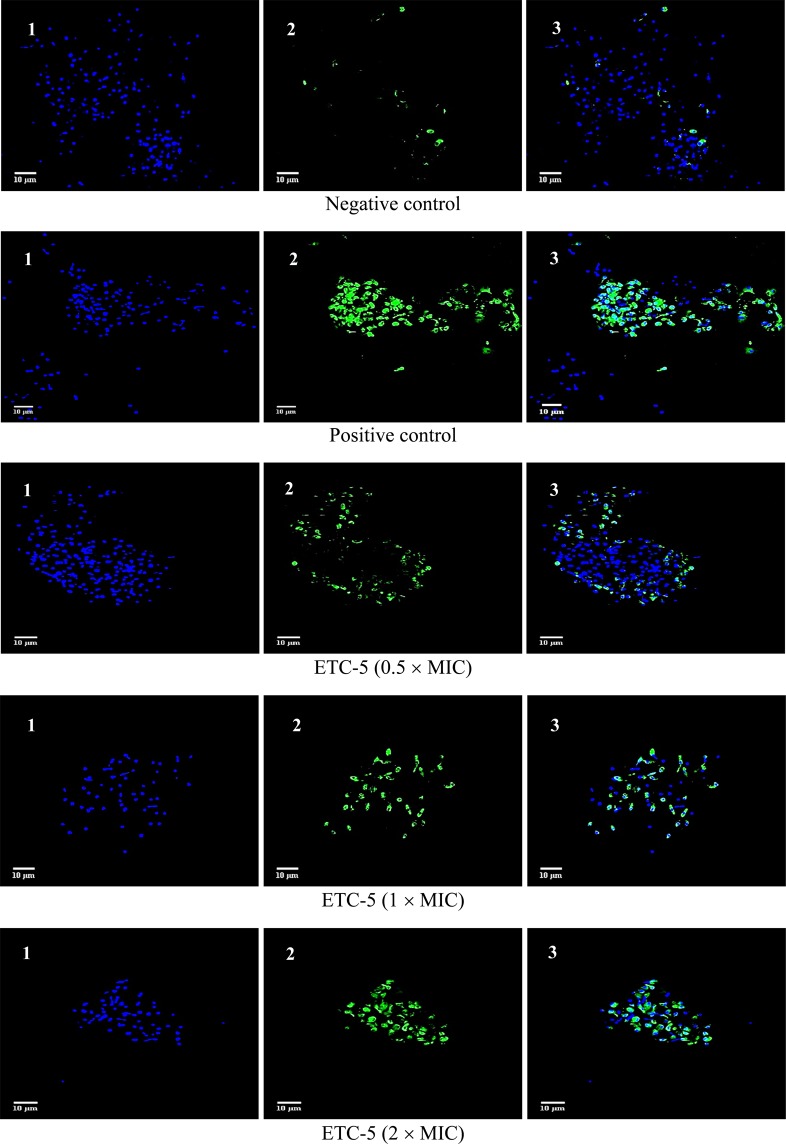
Confocal scanning fluorescence images of *C*. *albicans* SC5314 cells after exposed to 0.5 × MIC, 1 × MIC and 2 × MIC of ETC-5. 1, 2 and 3 represents live or dead intact cells stained with Hoechst 33342 dye (blue fluorescence), apoptotic cells indicating active DNA fragmentation stained with Alexa Fluor 488 (green fluorescence) and a merged image, respectively. Untreated cells with no apoptosis and cells exposed to H_2_O_2_ (250 μmol/l) were used as negative and positive controls respectively.

**Figure 5:**
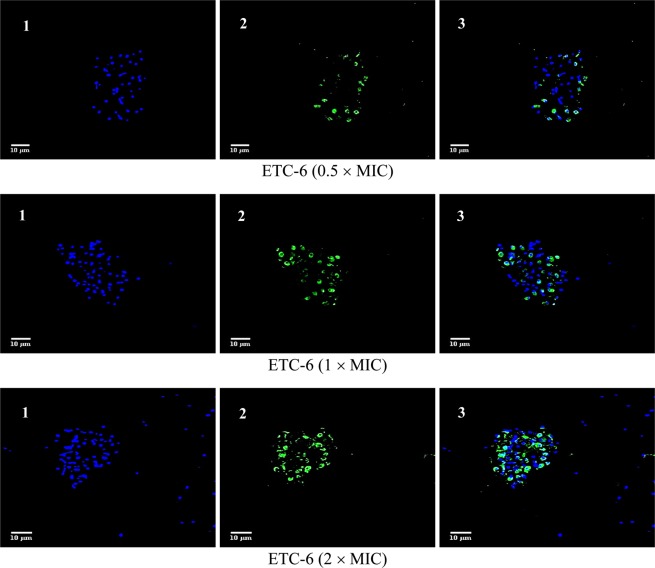
Confocal scanning fluorescence images of *C*. *albicans* SC5314 cells after exposed to 0.5 × MIC, 1 × MIC and 2 × MIC of ETC-6. 1, 2 and 3 represents live or dead intact cells stained with Hoechst 33342 dye (blue fluorescence), apoptotic cells indicating active DNA fragmentation stained with Alexa Fluor 488 (green fluorescence) and a merged image, respectively.

**Figure 6:**
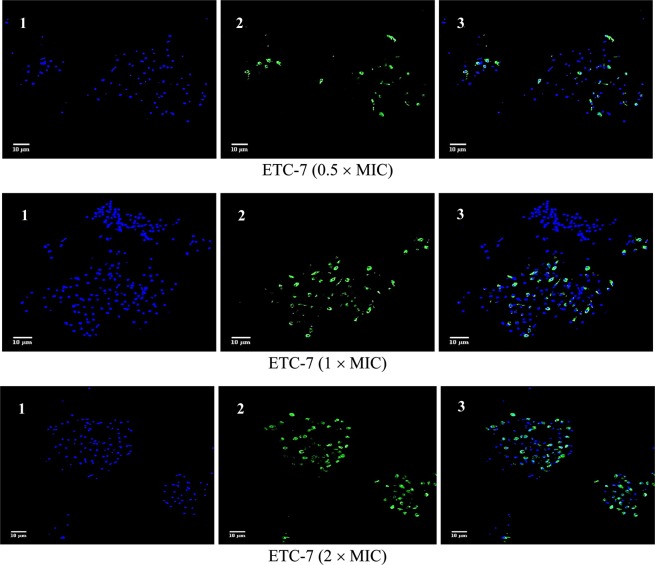
Confocal scanning fluorescence images of *C*. *albicans* SC5314 cells after exposed to 0.5 × MIC, 1 × MIC and 2 × MIC of ETC-7. 1, 2 and 3 represents live or dead intact cells stained with Hoechst 33342 dye (blue fluorescence), apoptotic cells indicating active DNA fragmentation stained with Alexa Fluor 488 (green fluorescence) and a merged image, respectively.

From the PS externalisation assay, it was evident that the active compounds showed prominent late apoptosis and therefore DNA fragmentation, which is a characteristic feature of late apoptosis, was studied. To study this hallmark of late apoptosis in response to these active compounds, terminal deoxynucleotidyl transferase-mediated dUTP nick end labelling (TUNEL) assay was used. The TUNEL assay is based on the incorporation of modified dUTP at the 3′-OH ends of fragmented DNA. For differentiation, cells were also counterstained with Hoechst 33342 dye (blue). *C*. *albicans* strains (fluconazole susceptible *C*. *albicans* 4175, fluconazole resistant *C*. *albicans* 5112 and a control strain *C*. *albicans* SC5314), when exposed to different concentrations (0.5 × MIC, 1 × MIC and 2 × MIC) of the test compounds ETC-5 (Figs. 4 and S4), ETC-6 (Figs. 5 and S4) and ETC-7 (Figs. 6 and S4) exhibited significant increase in proportion of TUNEL positive nuclei (green fluorescent spots) compared to the negative control (untreated cell population). The results also revealed that the effect was dose dependent as the number and intensity of green fluorescence of TUNEL positive nuclei increased when exposed to high concentrations of the test compounds. In concurrence with the results of other assays, ETC-5 was the most active compound against all the strains of *C*. *albicans* to induce DNA damage followed by ETC-6 and ETC-7. As expected, positive control (250 μmol/L H_2_O_2_) showed increased green fluorescence indicating higher number of late apoptotic cells (Fig. 4). These results advocated that the most active derivatives (ETC-5, ETC-6 and ETC-7) induced cell death through DNA damage in *C*. *albicans*.

#### Mitochondrial membrane potential (*Δψ*_*m*_)

**Figure 7:**
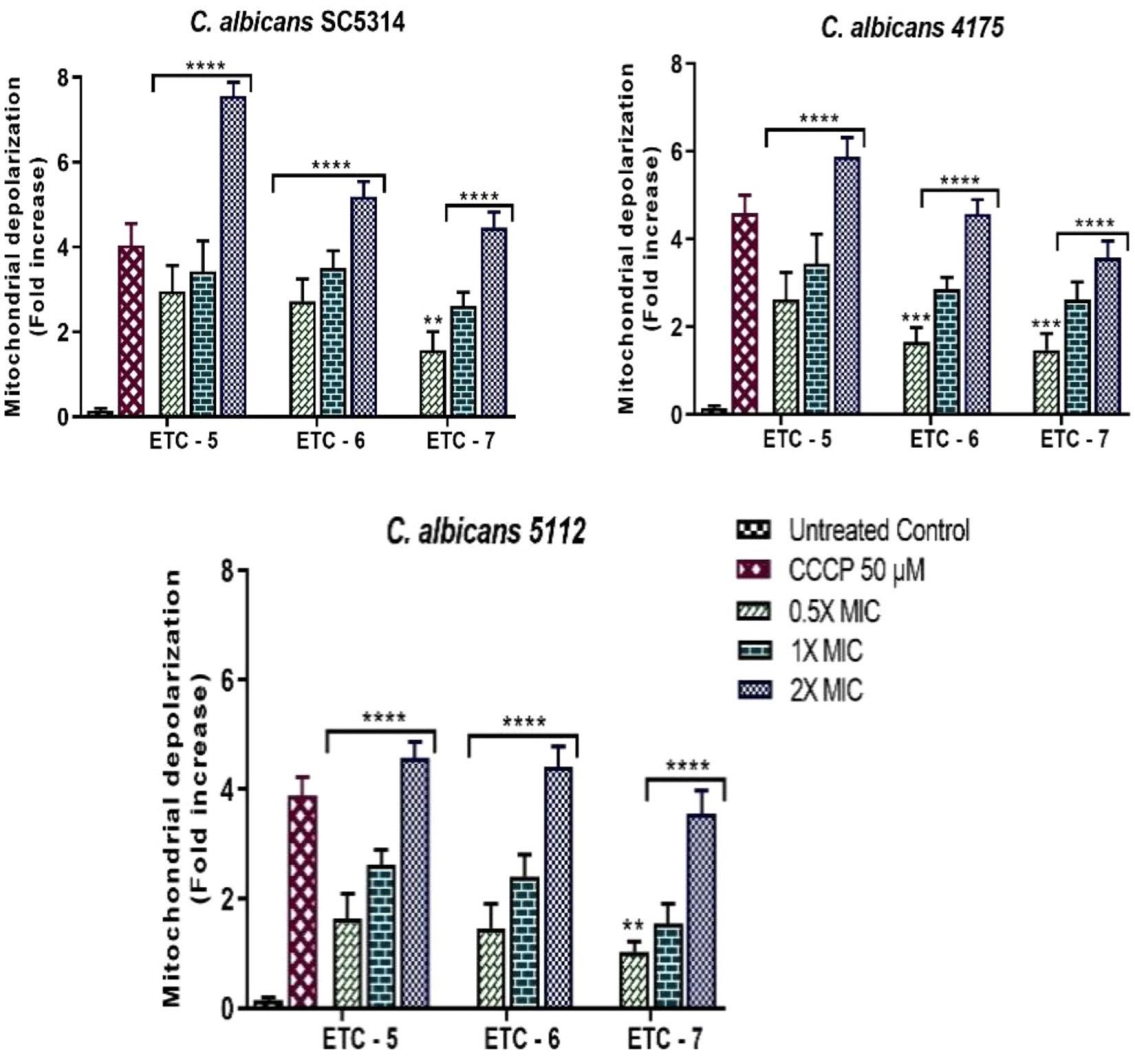
Fold increases in mitochondrial depolarization in ETC-5, ETC-6 and ETC-7 treated *C*. *albicans* SC5314, *C*. *albicans* 4175 and *C*. *albicans* 5112 in comparison to untreated control. 50 µM carbonyl cyanide 4-(trifluoromethoxy) phenylhydrazone (FCCP) treated cells were used as positive control. Data are presented from three independent experiments using means ± S.D. ****P < 0.0001, ***P: 0.0002. **P: 0.0012.

To measure the effect of these active compounds on the mitochondrial membrane potential (Δψ_m_), JC-10 dye was used. This dye aggregates in mitochondria of normal cells and produces red florescence, whereas in apoptotic cells with collapsed mitochondrial membrane potential it remains in the monomeric form and produces green florescence. In this study, after 3 h of exposure with test compounds (ETC-5, ETC-6 and ETC-7), significant decrease in mitochondrial membrane potential were observed in treated *C*. *albicans* cells in comparison to untreated control cells (Fig. 7). The results of this study showed that ETC-5, the most active compound decreased the mitochondrial membrane potential by 1.64–2.96, 2.63–3.44 and 4.58–7.57 folds at concentrations of 0.5 × MIC, 1 × MIC and 2 × MIC, respectively. ETC-6 and ETC-7 showed decrease in mitochondrial potential by 1.46–2.73, 2.41–3.52, 4.42–5.20 and 1.04–1.58, 1.56–2.62, 3.55–4.46 folds respectively at 0.5 × MIC, 1 × MIC and 2 × MIC. These results clearly indicated that the mitochondrial depolarization was dose dependent as there was a sharp increase in the ratio of red/green fluorescence intensity with increasing concentrations of the test compounds.

#### Cytochrome c oxidase activity

**Figure 8:**
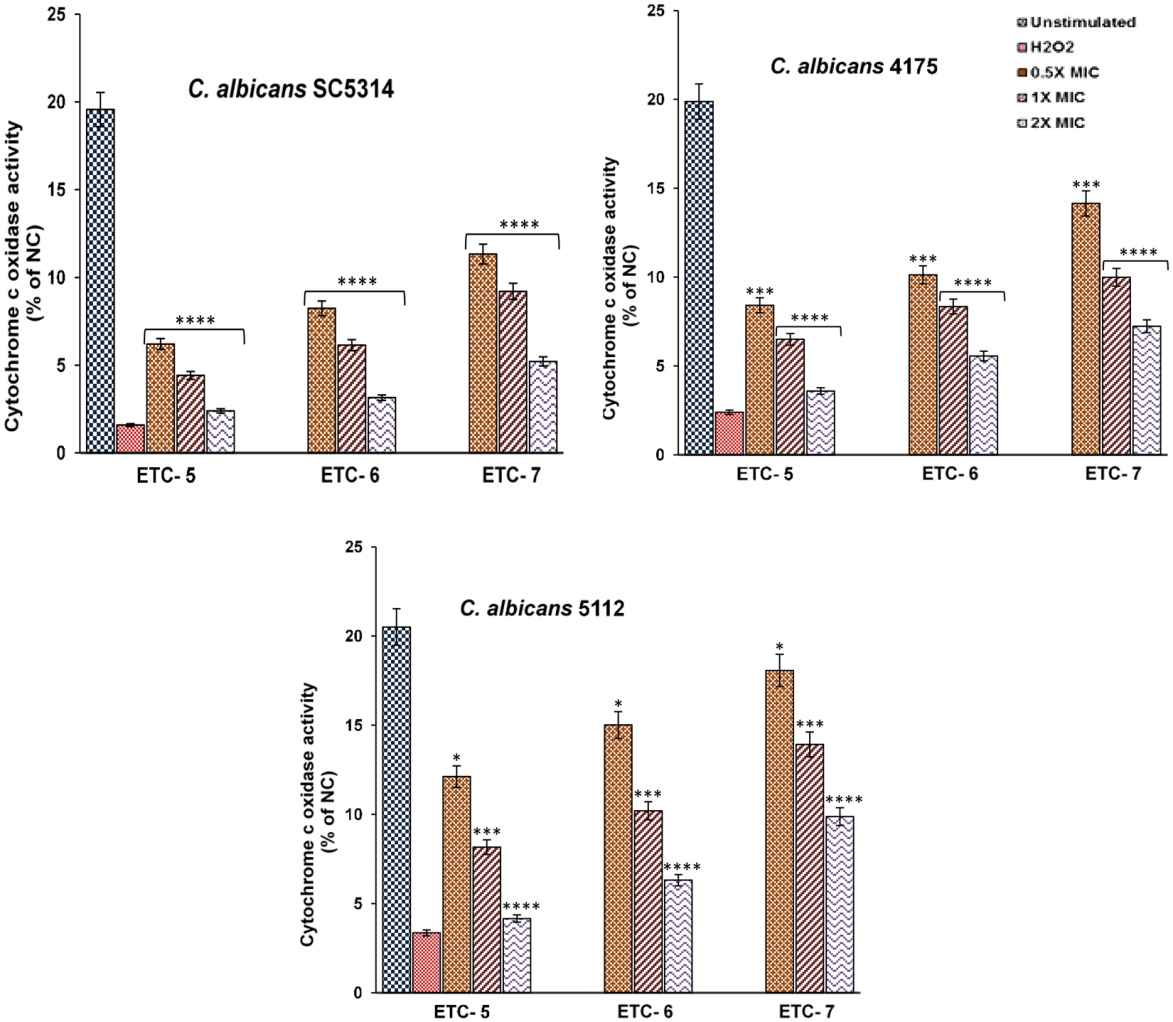
Cytochrome c oxidase activity in *C*. *albicans* cells, after exposed to varying concentrations of ETC-5, ETC-6 and ETC-7 for 3 h, exhibited significant decrease in enzyme activity in contrast to untreated control. Cells exposed to 250 μmol/l H_2_O_2_ were used as negative and positive controls, respectively. Data are presented from three independent experiments using means ± S.D. ****P < 0.0001, ***P: 0.0001. *P: 0.0104.

To further confirm the apoptotic characteristics of ETC-5, ETC-6 and ETC-7, effect of varying concentrations of these compounds on cytochrome c oxidase activity was studied in *C*. *albicans* SC5314, *C*. *albicans* 4175 and *C*. *albicans* 5112. A significant decrease in enzyme activity was observed in a dose dependent manner after exposure of cells to test compounds compared to the unstimulated control (Fig. 8). ETC-5, being the most active compound, decreased enzyme activity up to 6.20–12.11%, 4.42–8.16% and 2.39–4.16% at concentrations of 0.5 × MIC, 1 × MIC and 2 × MIC, respectively, while as these figures for ETC-6 and ETC-7 are 8.24–15.01%, 6.14–10.20% and 3.15–6.31% and 11.33–18.07%, 9.20–13.92% and 5.21–9.88%, respectively at 0.5 × MIC, 1 × MIC and 2 × MIC (Fig. 8). These results also demonstrated that the test compounds influence cytochrome c oxidase activity by drastically reducing the enzyme activity in different *C*. *albicans* cells.

### Cytotoxicity studies by haemolytic activity

**Figure 9:**
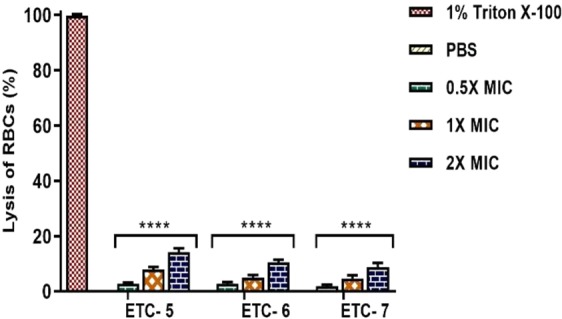
Cytotoxicity assay by haemolytic activity. ETC-5, ETC-6 and ETC-7 at varying concentrations showed significant difference in haemolysis of RBCs compared to positive control 1% Triton X-100 (reference for 100% haemolysis). Phosphate buffer saline was used as negative control, which showed complete absence of haemolysis. Data are presented from three independent experiments using means ± S.D. ****P < 0.0001.

Cytotoxicity of ETC-5, ETC-6 and ETC-7 at varying concentrations was checked by haemolytic assay using horse red blood cells (RBCs). When compared to positive control (Triton X-100), which causes 100% lysis, test compounds showed cell haemolysis in the range of 1.98%–15.18% (Fig. 9). At 2 × MIC values, ETC-5, ETC-6 and ETC-7 showed haemolytic activity of 15.18%, 10.23% and 8.91%, respectively. PBS was used as a negative control, which showed no lysis of RBCs. These results confirmed low cytotoxicity effect of the newly synthesized ETCs, suggesting that they are potentially safe to use.

## Discussion

The demand for finding new antifungals is a high medical priority which was stimulated by the emergence of MDR *Candida* species and resistant virulent forms. With the increasing interest of the use of medicinal plants and knowing the importance of medicinal chemistry, semi-synthetic natural compounds and derivatives have gained a tremendous attention to substitute or augment the current antimicrobial treatment regimens^21^. Modifying natural compounds to increase their efficacy, solubility, as well as to lower their side effects have been valued to discover novel drugs against various infectious diseases including candidiasis^22,23^.

Our previous findings on the antifungal activity of eugenol-tosylates encouraged us to synthesize some new eugenol-tosylates with different functional groups and make detailed studies of their antifungal profile. Preliminary antifungal susceptibility testing results showed that the test compounds under study (ETC-1–ETC-7) have potent antifungal activity against all the tested *C*. *albicans* isolates. Previously, we reported that ETCs impair ergosterol biosynthesis pathway in *Candida* cells, in the same manner as their precursor compound eugenol, resulting in the reduction of number of viable cells^5^. Eugenol has also been reported to cause apoptosis after reduction in ergosterol biosynthesis in *Candida* cells^24^. Therefore, in this study, we focussed on the antifungal mechanism of the three most active eugenol-tosylate congeners ETC-5, ETC-6 and ETC-7, from the preliminary studies, against *C*. *albicans* by studying apoptosis. The characteristic markers of apoptosis such as PS externalization, DNA fragmentation, mitochondrial depolarization and cytochrome c oxidase activity were used to investigate the pattern of cell death. From the results, it is evident that these compounds can induce *C*. *albicans* cell death *via* apoptosis and necrosis in a dose-dependent manner.

Compounds containing the sulfone (–SO_2_–) group in their ring skeleton are potent antimicrobial agents especially, dapsone and its derivatives which are effective against different bacteria^25^. Out of the seven derivatives used in this study, three were found to be strong antifungals, which is possibly due to the different substituents on the phenyl ring of sulfone moiety. Presence of a trifluoromethyl group in ETC-5, a trifluoromethoxy group in ETC-6, and a carboxylic acid group in ETC-7 at position-4 of the phenyl ring of the sulfone moiety sharply increased the antifungal activity of the derivatives compared to methyl substituted (ETC-1), unsubstituted (ETC-2), methoxy (ETC-3) and a mono-fluoro substituted phenyl ring in these derivatives. The difference in antifungal activity caused due to the presence of different substituents could be attributed to the different chemical nature of the substituents. In ETC-5 the presence of trifluoromethyl group renders significant antifungal activity to the molecule which is due to the unique chemical and physiological stability of this group^26,27^. The unique chemical and isosteric nature of this group is the reason that it is found in some notable drugs such as efavirenz, fluoxetine, celecoxib, and many more.

With the increasing drug resistance, paradigm shift of pharmaceutical companies from developing antifungals has also increased. Therefore, to accommodate drug discovery and to filter the process, new optimizing measure are gaining more interest. Among these measures, physicochemical parameters have been incorporated for the development of therapeutic agents. These physicochemical parameters also assess absorption, distribution, metabolism, excretion, and toxicological (ADMET) properties of test compounds^18^. These parameters play an important role and can easily avoid 50–60% failures in drug development^18^. After detailed research, it has been concluded that the test compounds should have a lipophilicity within a range of logD between ~ 1 and 3^16^ or ≤5^28^, solubility of >100 μM, and a logP value < 3.25. The other important parameter for drug discovery is oral bioavailability, good intestinal absorption and membrane permeability, which are also associated with logP, molecular weight (MW), or hydrogen bond acceptors and donors count in a molecule. In this study, all the test compounds obeyed the ‘Rule of Five and therefore, these molecules show desirable drug likeness properties which encourage their animal model studies.

Apoptosis in metazoans is essential for their homeostasis and maintenance^29^. There are numerous studies which reported apoptosis in yeast, induced by various agents including many natural compounds^30–33^. Our results demonstrated that *C*. *albicans* cells after being exposed to varying concentrations of ETCs exhibited dose-dependent effect on the key markers of yeast apoptosis such as PS externalization, DNA damage, mitochondrial depolarization and decrease in cytochrome c oxidase activity. In FITC annexin V/PI-staining, cells at sub-MIC values showed early apoptosis, while a necrosis was observed at higher concentrations of test compounds. Further, results from TUNEL assay corroborate the results observed with FITC annexin V/PI-staining and are in agreement with the previous findings where natural products were reported to cause dose dependent DNA fragmentation-based apoptosis in *Candida* cells^34^.

Mitochondria has been reported an important cell organelle to play significant role in the physiology of cell death^34^. Depolarization in mitochondria results in the release of cytochrome c and other pro-apoptotic factors into the cytosol^35,36^. The release of cytochrome c from the mitochondria into the cytosol directly correlates with a decrease in cytochrome c oxidase activity. After its release from the mitochondria into the cytosol, it leads to the activation of yeast metacaspase Yca1p (the only caspase which play an essential role in yeast apoptosis and an ortholog of mammalian caspases), which in turn results in the activation of caspase cascade inducing apoptosis. This is observed most commonly with such forms of cell death in yeast^37^. In this study, decrease in cytochrome c oxidase activity and mitochondrial depolarization were measured to understand the effect of test compounds on the intrinsic pathway. Due to the lipophilic nature (cLogP = 3.87–5.24) of these compounds it is important to focus on the intrinsic pathway over extrinsic pathway, which are classical pathways to operate apoptosis. Our results demonstrated significant mitochondrial depolarization and decrease in cytochrome c oxidase activity in *C*. *albicans* cells after being exposed with test compounds at different concentrations. At higher concentrations, there are evidences of necrosis which could be the reason of rapid and irreversible antifungal activity of these compounds and can be correlated to the structural and functional damage of membranes.

High toxicity of the currently used drugs is one of the major hindrances to use them in immunocompromised patients. Therefore, it is important to find new drugs that allow specific manipulation of cell death without costing human counterparts. ETCs significantly showed potent antifungal activity and induced apoptosis in *C*. *albicans*, thus it is important to check the cytotoxicity of these test compounds. Haemolytic assay, using horse RBCs, was used to gauge *in vivo* toxicity of these compounds to host cells. The results indicated that these ETCs were significantly less toxic when compared to triton X-100, which advocates its safe *in vivo* use for future animal studies.

In conclusion, these results showed that three derivatives (ETC-5, ETC-6 and ETC-7) out of the seven eugenol tosylate congeners synthesized have potent antifungal activity and induced apoptosis in *C*. *albicans*. The mitochondrial depolarization and release of cytochrome c into cytosol supported apoptosis induction in *C*. *albicans* cells via metacaspase-dependent apoptotic pathway. To the best of our knowledge, this study is the first to assess the physiology and mode of cell death in *C*. *albicans* caused by ETCs. However, further studies are required to facilitate the development of novel antifungal drugs from these compounds.

## Materials and Methods

### Synthesis

All the compounds ETC-1–ETC-7 were synthesized from 2-methoxy-4-(prop-2-en-1-yl)phenol (1) as reported in our previous study^5^.

#### 2-methoxy-4-(prop-2-en-1-yl) phenyl-4-methylbenzenesulfonate (ETC-1)

Yield: 85%; Anal. Calc. for C_17_H_18_O_4_S; C, 64.13; H, 5.70%, found; C, 64.28; H, 5.56%; IR_max_ cm^−1^: 3033 (C–H stretch), 1587 (C=C, Ar), 1315, 1158 (S=O); ^1^H NMR (DMSO-*d*_6_) (ppm): 7.87–7.59 (4H, m, Ar-H), 6.68–6.52 (3H, m, Ar-H), 6.25 (1H, m), 4.86 (1H, dd, J = 15.2 Hz, 6.8 Hz), 4.48 (1H, dd, J = 15.2 Hz, 6.8 Hz), 3.90 (2H, d, CH_2_), 3.54 (3H, s, OCH_3_), 2.25 (3H, s, CH_3_); ^13^CNMR (DMSO-*d*_6_) (ppm): 148.9, 144.6, 138.5, 137.0, 135.4, 133.5, 130.6, 129.3, 128.0, 122.5, 118.5, 115.7, 56.4, 49.4, 24.8; ESI-MS m/z [M + H]^+^ 319.10; [M + Na]^+^ 342.06.

#### 2-methoxy-4-(prop-2-en-1-yl)-phenylbenzenesulfonate (ETC-2)

Yield: 84%; Anal. Calc. for C_16_H_16_O_4_S; C, 63.14; H, 5.30%, found; C, 63.30; H, 5.18%; IR_max_ cm^−1^: 3025 (C–H stretch), 1595 (C=C, Ar), 1308, 1150 (S=O); ^1^H NMR (DMSO-*d*_6_) (ppm): 7.95–7.50 (4H, m, Ar-H), 7.35 (1H, t, J = 7.8 Hz), 6.84–6.65 (3H, m, Ar-H), 6.22 (1H, m), 4.85 (1H, dd, J = 15.2 Hz, 6.8 Hz), 4.45 (1H, dd, J = 15.2 Hz, 6.8 Hz), 3.85 (2H, d, CH_2_), 3.68 (3H, s, OCH_3_), ^13^CNMR (DMSO-*d*_6_) (ppm): 149.8, 143.5, 138.6, 136.2, 134.6, 131.2, 130.1, 129.6, 128.7, 122.8, 118.9, 115.3, 58.6, 45.6; ESI- MS m/z [M + H]^+^ 305.10.

#### 2-methoxy-4-(prop-2-en-1-yl) phenyl-4-methoxylbenzenesulfonate (ETC-3)

Yield: 75%; Anal. Calc. for C_17_H_18_O_5_S; C, 61.06; H, 5.43%, found; C, 61.20; H, 5.49%; IR_max_ cm^−1^: 3030 (C–H stretch), 1583 (C=C, Ar), 1311, 1155 (S=O); ^1^H NMR (DMSO-*d*_6_) (ppm): 7.87–7.59 (4H, m, Ar-H), 6.68–6.52 (3H, m, Ar-H), 6.25 (1H, m), 4.86 (1H, dd, J = 15.2 Hz, 6.8 Hz), 4.48 (1H, dd, J = 15.2 Hz, 6.8 Hz), 3.90 (2H, d, CH_2_), 3.78 (3H, s, OCH_3_), 3.54 (3H, s, OCH_3_); ^13^CNMR (DMSO-*d*_6_) (ppm): 148.5, 145.2, 138.1, 137.2, 135.3, 133.4, 131.5, 129.1, 128.0, 121.8, 118.2, 114.8, 56.8, 55.6, 46.8; ESI-MS m/z [M + H]^+^ 335.10.

#### 2-methoxy-4-(prop-2-en-1-yl) phenyl-4-fluorobenzenesulfonate (ETC-4)

Yield: 70%; Anal. Calc. for C_16_H_15_O_4_FS; C, 59.62; H, 4.59%, found; C, 59.54; H, 4.50%; IR_max_ cm^−1^: 3020 (C–H stretch), 1572 (C=C, Ar), 1380 (C-F stretch), 1315, 1158 (S=O stretch); ^1^H NMR (DMSO-d6) (ppm): 7.84–7.68 (4H, m, Ar-H), 6.68–6.41 (3H, m, Ar-H), 6.22 (1H, m), 4.86 (1H, dd, *J* = 15.6 Hz, 6.2 Hz), 4.48 (1H, dd, *J* = 15.6 Hz, 6.2 Hz), 3.90 (2H, d, CH_2_), 3.65 (3H, s, OCH_3_); ^13^CNMR (DMSO-*d*_6_) (ppm): 155.1, 148.2, 145.5, 143.4, 138.1, 136.3, 133.4, 128.9, 122.1, 118.2, 116.0, 115.2, 56.5, 48.5; ESI-MS m/z [M + H]^+^ 323.08.

#### 2-methoxy-4-(prop-2-en-1-yl) phenyl-4-trifluoromethylbenzenesulfonate (ETC-5)

Yield: 68%; Anal. Calc. for C_17_H_15_O_4_F_3_S; C, 54.83; H, 4.06%, found; C, 54.89; H, 4.20%; IR_max_ cm^−1^: 3022 (C–H stretch), 1574 (C=C, Ar), 1385 (C-F stretch), 1310, 1150 (S=O stretch); ^1^H NMR (DMSO-*d*_6_) (ppm): 7.90–7.72 (4H, m, Ar-H), 6.88–6.65 (3H, m, Ar-H), 6.30 (1H, m), 4.80 (1H, dd, J = 15.6 Hz, 6.2 Hz), 4.45 (1H, dd, J = 15.6 Hz, 6.2 Hz), 3.87 (2H, d, CH_2_), 3.65 (3H, s, OCH_3_);^13^CNMR (DMSO-d6) (ppm): 155.4, 148.6, 145.4, 143.0, 138.2, 136.5, 133.1, 128.5, 126.0, 122.0, 118.5, 116.2, 115.5, 56.5, 48.2; ESI-MS m/z [M + H]^+^ 373.10.

#### 2-methoxy-4-(prop-2-en-1-yl) phenyl-4-trifluoromethoxylbenzenesulfonate (ETC-6)

Yield: 68%; Anal. Calc. for C_17_H_15_O_5_F_3_S; C, 52.58; H, 3.89%, found; C, 52.50; H, 3.92%; IR_max_ cm^−1^: 3022 (C–H stretch), 1580 (C=C, Ar), 1380 (C-F stretch), 1315, 1155 (S=O stretch); ^1^H NMR (DMSO-d6) (ppm): 7.95–7.78 (4H, m, Ar-H), 6.98–6.72 (3H, m, Ar-H), 6.34 (1H, m), 4.85 (1H, dd, J = 15.6 Hz, 6.2 Hz), 4.40 (1H, dd, J = 15.6 Hz, 6.2 Hz), 3.89 (2H, d, CH_2_), 3.66 (3H, s, OCH_3_); ^13^CNMR (DMSO-d6) (ppm): 156.0, 148.6, 145.5, 143.5, 138.5, 136.5, 134.0, 128.8, 126.0, 122.1, 118.1, 116.2, 115.0, 56.3, 48.0; ESI-MS m/z [M + H]^+^389.07.

#### 4-{[2-methoxy-4-(prop-2-en-1-yl) phenoxy]sulfonyl}benzoic acid (ETC-7)

Yield: 68%; Anal. Calc. for C_17_H_16_O_6_S; C, 58.61; H, 4.63%, found; C, 58.75; H, 4.80%; IR_max_ cm^−1^: 3022 (C-H stretch),2971 (OH stretch), 1725 (C=O stretch), 1574 (C=C, Ar), 1310, 1150 (S=O stretch); ^1^H NMR (DMSO-d6) (ppm): 9.15 (s, 1H, OH), 7.90–7.76 (4H, m, Ar-H), 6.92–6.70 (3H, m, Ar-H), 6.32 (1H, m), 4.85 (1H, dd, J = 15.4 Hz, 6.4 Hz), 4.45 (1H, dd, J = 15.4 Hz, 6.4 Hz), 3.87 (2H, d, CH_2_), 3.62 (3H, s, OCH_3_); ^13^CNMR (DMSO-d6) (ppm): 168.7 (C=O), 154.4, 148.0, 145.1, 142.5, 138.0, 136.5, 135.1, 128.3, 126.7, 122.0, 118.0, 116.0, 115.6, 56.0, 48.1; ESI-MS m/z [M + H]^+^ 349.08.

### Fungal strains and growth conditions

In this study, 10 clinical *Candida albicans* isolates, comprising of 5 fluconazole susceptible and 5 fluconazole resistant, were used. *C*. *albicans* SC5314 was also used as a control laboratory strain. Clinical strains used in this study were stored at −80 °C supplemented with 20% glycerol in the Department of Clinical Microbiology and Infectious Diseases, University of the Witwatersrand, Johannesburg and were previously collected under the ethical clearance number M000402, obtained from the Human Research Ethics Committee, University of the Witwatersrand. For experimental purpose each strain from glycerol stock was recovered on Sabouraud Dextrose (SDA) agar plates at 37 °C for 24 h.

### Antifungal susceptibility testing

Minimum Inhibitory Concentration (MIC) and Minimum Fungicidal Concentrations (MFC) of all newly synthesized eugenol-tosylate congeners (ETC-1–ETC-7) against *C*. *albicans* strains were determined by broth microdilution assay in accordance with the approved guidelines described by Clinical and Laboratory Standards Institute (CLSI) guidelines M27-A^38^. The test compounds were prepared in 1% DMSO to a stock concentration of 8192 µg/ml. Two-fold dilutions of test compounds (100 µL) were prepared in 96-well flat-bottom microtiter plates, inoculated with 100 µl of inoculum (1 × 10^6^ cells/mL) and incubated at 37 °C for 24 hours. In every set of experiment, positive control (fluconazole), negative vehicle control (1% DMSO) and culture control (media and cells only) were included. MIC’s were determined visibly as the lowest concentration of the test compounds that inhibited growth of test organism. To determine MFC, each well without visible growth was sub-cultured onto agar plates. Concentration in the first well with no growth on plate was taken as MFC. On the basis of MICs values, most active compounds were selected and used for further studies. The experiment was performed in triplicate to validate the results.

### Apoptosis

#### Protoplast preparation

To determine the physiology and mode of cell death in response to three most active compounds expression of apoptotic markers was analysed. To study the effect of test compounds on apoptotic markers protoplasts were prepared as described previously^39^, with modifications. Briefly, mid-exponential phase cells were harvested and exposed to various concentrations of test compounds (0.5 × MIC, 1 × MIC and 2×MIC) for 3 h. The cells were then washed twice with protoplast buffer- I (1 M sorbitol, 50 mM tris base, 10 mM MgCl_2_ and 30 mM DTT) and incubated in the same buffer for 15 minutes at 25 °C, followed by centrifugation at 1500 rpm for 5 minutes. Supernatant was removed and cells were incubated with buffer- II (1 M sorbitol, 50 mM tris base, 10 mM MgCl_2_ and 1 mM DTT) supplemented with lyticase enzyme (≥1 µg/mL) used to digest the cell wall for 1 h at 25 °C. Buffer- II were removed by centrifugation and then cells were incubated with buffer- III (1 M sorbitol, 50 mM tris base and 10 mM MgCl_2_) for 20 minutes at room temperature. After the incubation cells were again centrifuged at 1500 rpm for 5 minutes and supernatant was discarded. Protoplasts then finally washed once with PBS and re-suspended in the same for further use. In each washing steps the volume of these protoplast buffers were 5 times to cell volume with pH 7.4.

#### FITC annexin V/PI-staining

This assay was used to identify early apoptosis by detecting movement of phosphatidylserine (PS) from the inner to the outer leaflet of plasma membrane. FITC annexin V Apoptosis Detection Kit I (BD Biosciences, CA, USA) was used according to the manufacturer’s instructions. Protoplasts of *C*. *albicans* cells treated with different concentrations of the test compounds, as mentioned in above section, were resuspended in 1X annexin V binding buffer (0.1 M Hepes/NaOH pH 7.4, 1.4 M NaCl, 25 mM CaCl_2_) at a concentration of 1 × 10^6^ cells per ml. To the 100 µl of this solution, 5 µl each of FITC annexin V and propidium iodide (PI) was added. Tubes were gently vortexed and incubated for 15 minutes at 25 °C in the dark. After incubation, 400 µl of 1X annexin V binding buffer was added to each tube and analyzed using the BD LSRFortessa Flow cytometer (Becton Dickinson, NJ, USA). The cell populations in four different quadrants of a quadrant dot plot were analyzed and interpreted as; Q1: necrosis (annexin V^−^/PI^+^), Q2: late apoptosis (annexin V^+^/PI^+^), Q3: early apoptosis (annexin V^+^/PI^−^) and Q4: viable cells (annexin V^−^/PI^−^), by using software FlowJo_V10.

#### DNA damage

TUNEL assay was used to detect the DNA damage, a hallmark for late stages of apoptosis. Click-iT Plus TUNEL assay kit (Thermo Fisher scientific, MA, USA) was used according to the manufacturer’s instructions. Briefly, cells after exposed to different concentrations of test compounds were fixed in 4% paraformaldehyde then incubated for 15 minutes at room temperature, followed by protoplast preparation as described above. Protoplasts were then incubated with permeabilization reagent (0.25% Triton X-100 in PBS) for 20 minutes at room temperature, then rinsed twice with deionized water. TdT reaction buffer (100 µl) were added to each sample and incubated for 10 minutes at 37 °C. After incubation TdT reaction buffer were removed followed by addition of 50 µl TdT reaction mixture and incubated for 1 h at 37 °C in a humidified chamber. Protoplasts were then washed twice with 3% BSA in PBS for 5 minutes each, followed by immediate addition of Click-iT Plus TUNEL reaction cocktail (50 µl) to each sample and incubated for 30 minutes at 37 °C in the dark. After incubation reaction cocktail was removed and protoplasts were washed once with 3% BSA in PBS for 5 minutes. Cells were counterstained with Hoechst 33342 dye (Thermo Fisher scientific, MA, USA), by adding 100 µl of 1X Hoechst 33342 to each sample and incubated in dark for 15 minutes at room temperature followed by washing twice with PBS. Slides were examined by fluorescence microscopy using Zeiss Laser Scanning Confocal Microscope (LSM) 780 (Carl Zeiss Microscopy, Jena, Germany) with an excitation wavelength of 495 nm and emission wavelength of 519 nm. For Hoechst 33342 dye excitation wavelength of 350 nm and emission wavelength of 461 nm was used. Images were further analyzed by using software ImageJ.

#### Mitochondrial membrane potential (Δψ_m_)

For the detection of mitochondrial membrane potential changes in cells exposed with different concentrations of active tosylate congeners, the JC-10 mitochondrial membrane potential assay kit–microplate (abcam, Cambridge, UK) was used according to the manufacturer’s instructions. Protoplasts were prepared as described above at a concentration of 1 × 10^6^ cells/ml. 90 µl of this suspension was added to each well in 96-well microplate (with black wall and clear bottom) followed by addition of 50 µl/well JC-10 dye-loading solution. The dye loaded plate was incubated at 37 °C for 1 hour in dark. After incubation 50 µl/well assay buffer B was added followed by centrifugation at 800 × rpm for 2 minutes. Fluorescence intensity were monitored at Ex/Em = 490/525 nm and 540/590 nm with a BioTek Synergy HT microplate reader (BioTek Instruments, WA, USA) for ratio analysis. The ratio between aggregate (Em = 525 nm) and monomeric forms (Em = 590 nm) of JC-10, were measured to find the change in mitochondrial membrane potential. Increasing ratios indicate mitochondrial membrane depolarization. An untreated sample and a 50 µM carbonyl cyanide 4-(trifluoromethoxy) phenylhydrazone (FCCP) (Sigma – Aldrich St. Louis, MO, U.S.A.) treated sample were used as negative and positive controls, respectively.

#### Cytochrome c oxidase activity

After the cells were exposed to different concentrations of test compounds as mentioned above, mitochondria of the cells were isolated using Yeast Mitochondria Isolation Kit- MITOISO3 (Sigma – Aldrich St. Louis, MO, U.S.A.). Cytochrome c oxidase assay kit (ScienCell, Cat. No. 8278, CA, USA) was used to detect the cytochrome c oxidase activity. In all procedures, kits were used according to the manufacturer’s instructions. Briefly, prepared spheroplasts were followed by homogenization to release the mitochondria. Homogenate was centrifuged in 1X storage buffer (1 ml) at 6,500 × g for 10 minutes at 2–8 °C. Discard the supernatant and resuspend the final pellet in 200 µl of 1X storage buffer. Protein concentration was determined by using TCA Lowry method^40^. For the determination of cytochrome c oxidase activity, a reaction mixture was prepared (assay Buffer 940 µl, n-dodecyl β-D-maltoside solution 10 µl and mitochondrial protein 2 μg) in 1 ml cuvette followed by addition of 50 µl cytochrome c substrate solution. Immediately change in absorbance of cytochrome c was measured at 550 nm on a kinetic program (duration 30 seconds, interval 5 seconds) by using UV-1800 SHIMADZU spectrophotometer (Shimadzu Corporation, Japan).

### Cytotoxicity studies

The cytotoxicity of the test compounds was determined on horse red blood cells (NHLS, Johannesburg, South Africa) by testing the haemolytic activity of these compounds using the method described previously^41^, with some modifications. Briefly, in a sterile 15 ml falcon tube 5 ml of horse blood was centrifuged at 2000 × rpm for 10 minutes. The supernatant was discarded, and the cell pellet was washed three times with chilled PBS solution. The final pellet was used to yield a 10% RBC suspension with chilled PBS. The 10% suspension were further diluted to 1:10 in PBS, then 100 µl of this suspension was added to different concentration of test compounds (0.5 × MIC, 1 × MIC and 2 × MIC) in 1.5 ml tubes. The tubes were then incubated for 60 minutes at 37 °C followed by centrifugation at 2000 × rpm for 10 minutes. From each tube, 200 µl of the supernatant was transferred to a flat-bottomed 96-well microtiter plate and absorbance was monitored at 450 nm spectrophotometrically with an iMark microplate reader (Bio-Rad laboratories, CA, USA). Triton X-100 (1%) and PBS were used as positive and negative controls respectively. The percentage of haemolysis was calculated by the following equation:

$$ \% \,{\rm{haemolysis}}=\left[\frac{{\rm{A}}\,450\,{\rm{of}}\,{\rm{test}}\,{\rm{compound}}\,{\rm{treated}}\,{\rm{sample}}-{\rm{A}}\,450\,{\rm{of}}\,{\rm{buffer}}\,{\rm{treated}}\,{\rm{sample}}}{{\rm{A}}\,450\,\text{of}\,\,1 \% \,{\rm{Triton}}\,{\rm{X}}-100\,{\rm{treated}}\,{\rm{sample}}-{\rm{A}}\,450\,{\rm{of}}\,{\rm{buffer}}\,{\rm{treated}}\,{\rm{sample}}}\right]\times 100 \% $$

### Statistical analysis

All experiments were performed independently in triplicates (n=3), and data were presented as mean ± standard deviation (SD). The statistical analysis was performed using Dunnett’s multiple comparisons of a Two-way ANOVA test by GraphPad Prism software, version 8.0.1. Values of *P*(****P < 0.0001; ***P: 0.0001, 0.0002; **P: 0.0012; *P: 0.0104) were considered as statistically significant.

### Ethics approval and consent to participate

This study was approved by the Human Research Ethics Committee of University of the Witwatersrand (Johannesburg, South Africa). Existing stock cultures of *Candida albicans* used in this study were stored in the department of Clinical Microbiology and Infectious Diseases, University of the Witwatersrand, Johannesburg, South Africa.

## Supplementary information

Supplementary data.

## Data Availability

The data that support the findings of this study are available from the corresponding author upon request.
