# Supplementary material for: Cellular apoptosis and necrosis as therapeutic targets for novel Eugenol Tosylate Congeners against Candida albicans
Source: Sci Rep. 2020 Jan 27;10:1191. doi: 10.1038/s41598-020-58256-4 (PMC6985109; doi:10.1038/s41598-020-58256-4)
Supplement: Supplementary file 1 — Supplementary data. [file 41598_2020_58256_MOESM1_ESM.docx]

**Cellular apoptosis and necrosis as therapeutic targets for novel Eugenol Tosylate Congeners against *Candida albicans***

**Shabir Ahmad Lone^1^,** [**Mohmmad Younus Wani**](https://pubs.rsc.org/en/results?searchtext=Author%3AMohmmad%20Younus%20Wani)**^2^, Pascaline Fru^3^, Aijaz Ahmad^1,4*^**

^1^Department of Clinical Microbiology and Infectious Diseases, School of Pathology, Faculty of Health Sciences, University of the Witwatersrand, South Africa

^2^Chemistry Department, Faculty of Science, University of Jeddah, P.O. Box 80327, Jeddah 21589, Kingdom of Saudi Arabia

^3^Department of Surgery, School of Clinical Medicine, Faculty of Health Sciences, University of the Witwatersrand, South Africa

^4^Infection Control, Charlotte Maxeke Johannesburg Academic Hospital National Health Laboratory Service, South Africa.

**ORCID**:

[**https://orcid.org/0000-0003-2845-0727**](https://orcid.org/0000-0003-2845-0727) **(Aijaz Ahmad)**

[**https://orcid.org/0000-0002-1838-1337**](https://orcid.org/0000-0002-1838-1337) **(Mohmmad Y. Wani)**

[**https://orcid.org/0000-0003-1850-9625**](https://orcid.org/0000-0003-1850-9625) **(Shabir Lone)**

^*^Author for correspondence: Tel: +27 11 717 2515; Fax: +2786 295 3697

Email: [Aijaz.Ahmad@wits.ac.za](mailto:Aijaz.Ahmad@wits.ac.za); [Aijaz.Ahmad@nhls.ac.za](mailto:Aijaz.Ahmad@nhls.ac.za)

**^13^CNMR Spectra ETC-1 – ETC-7**


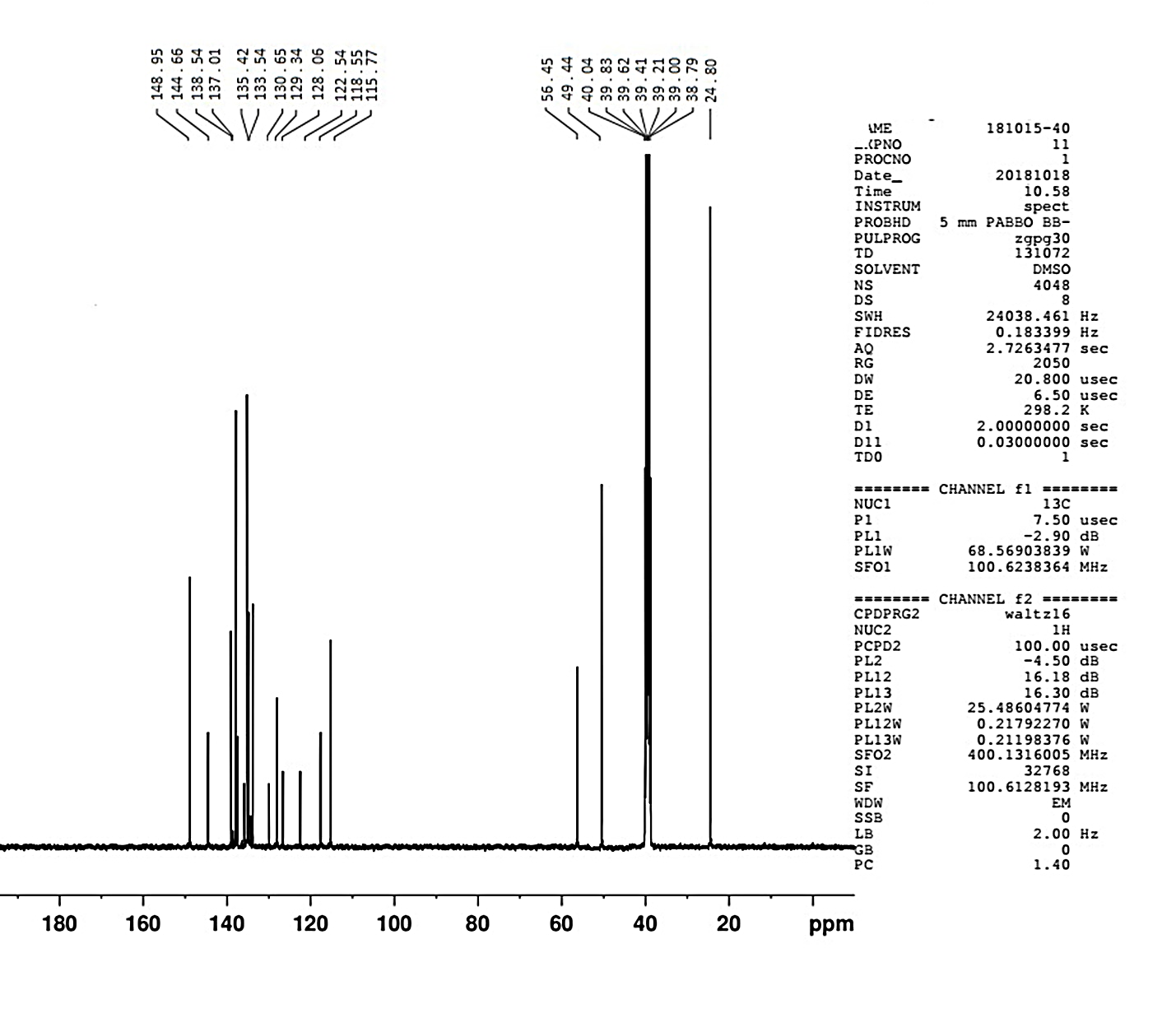


**^13^C NMR ETC-1**


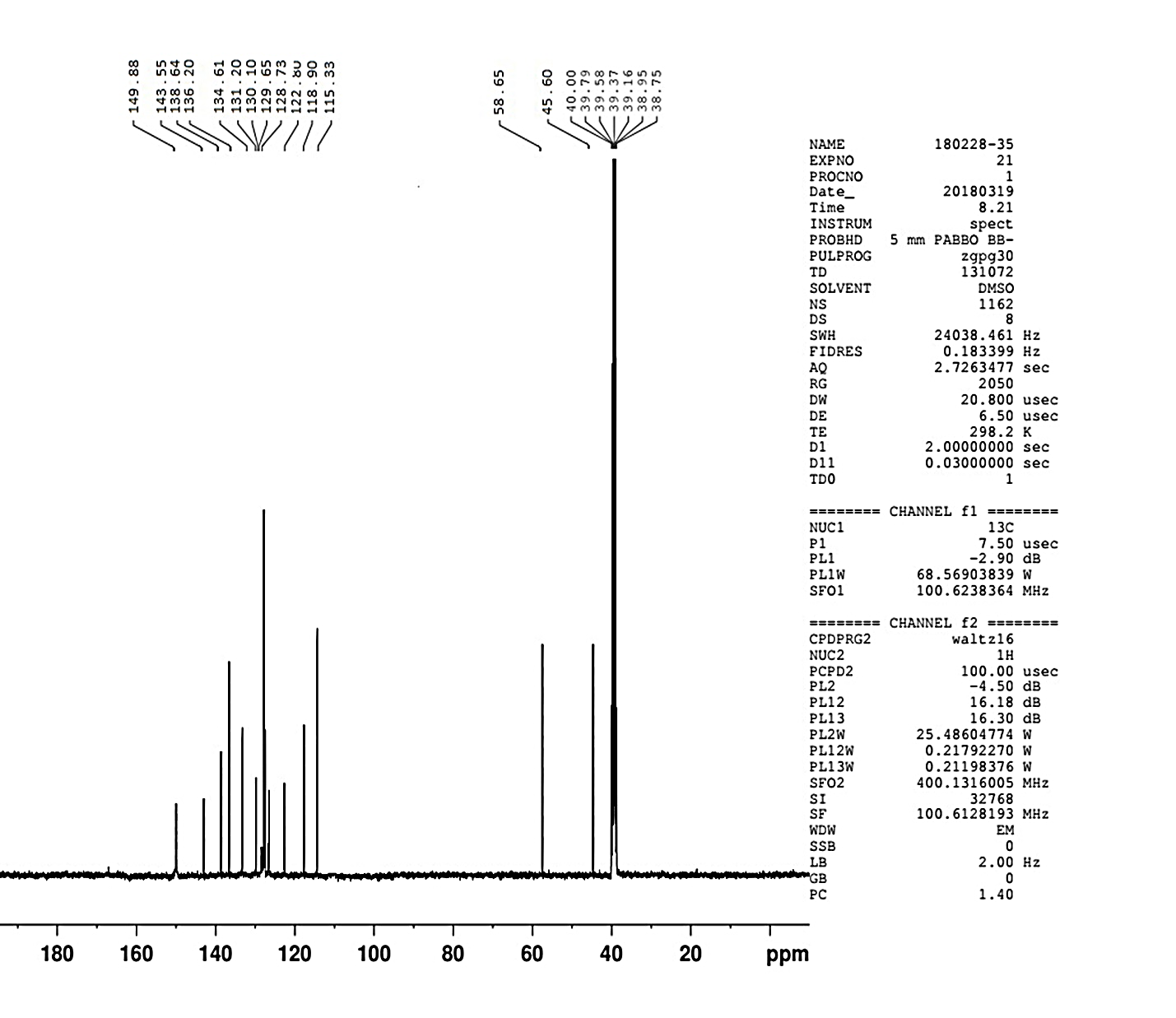


**^13^C NMR ETC-2**


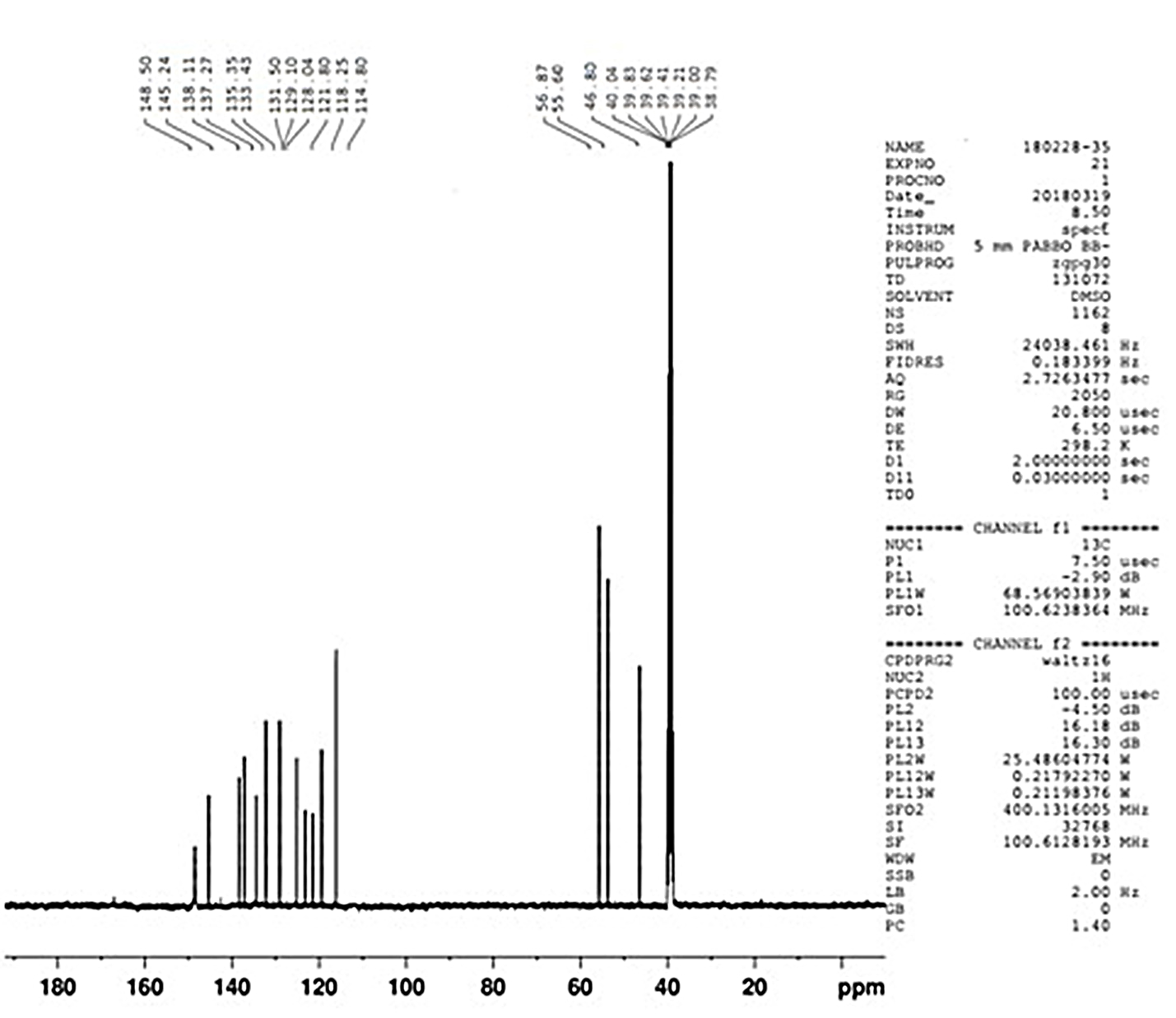


**^13^C NMR ETC-3**


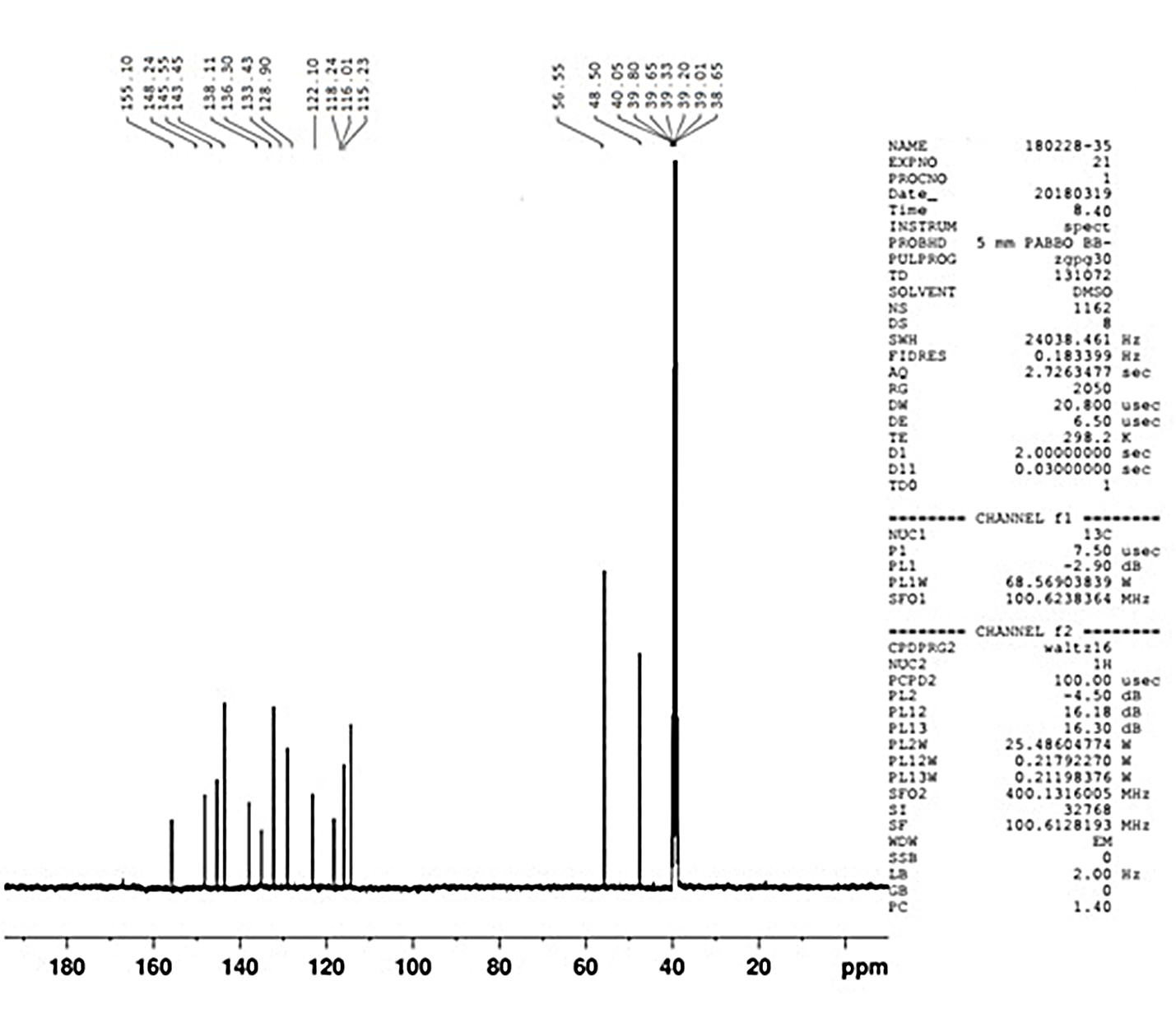


**^13^C NMR ETC-4**


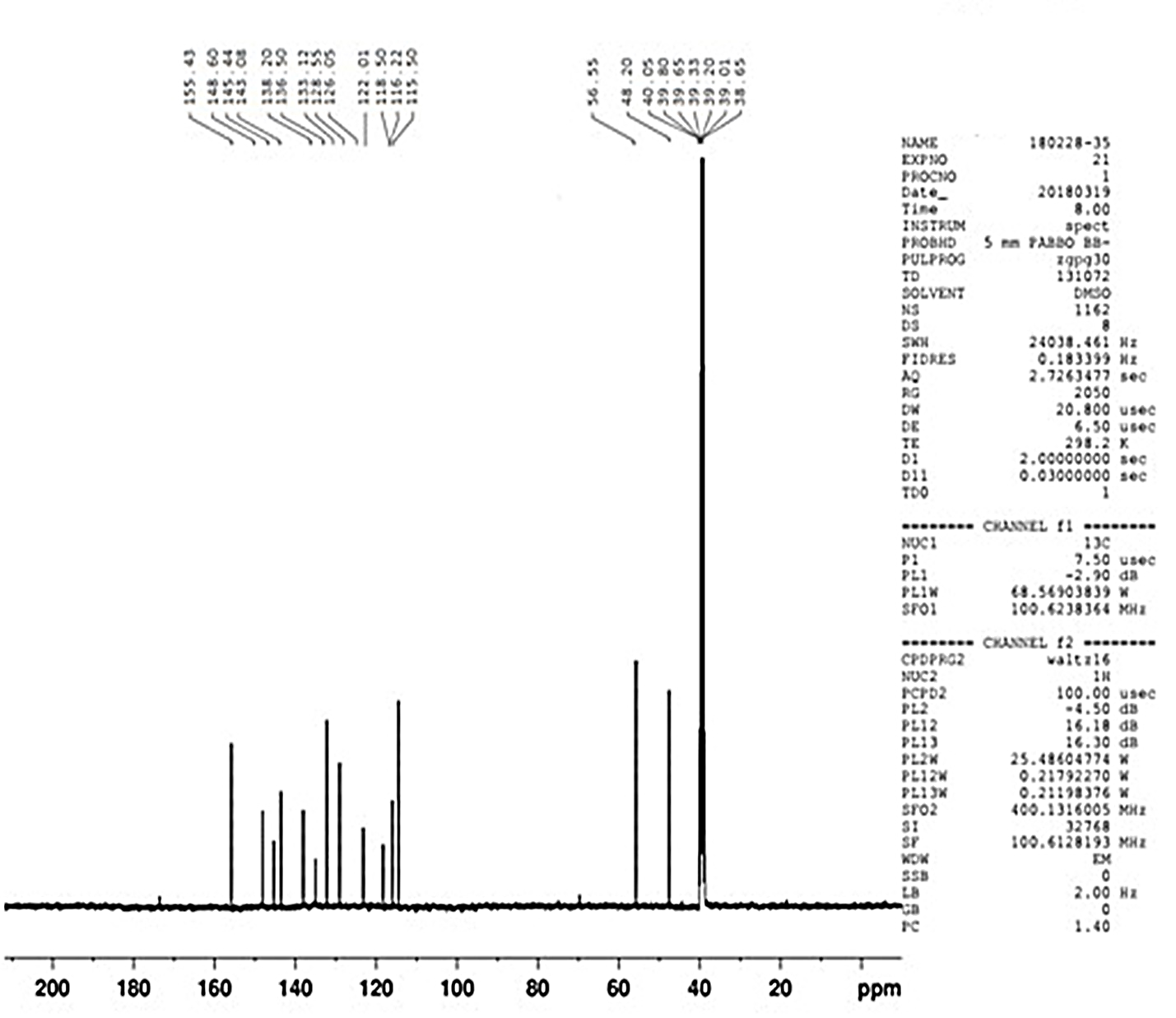


**^13^C NMR ETC-5**


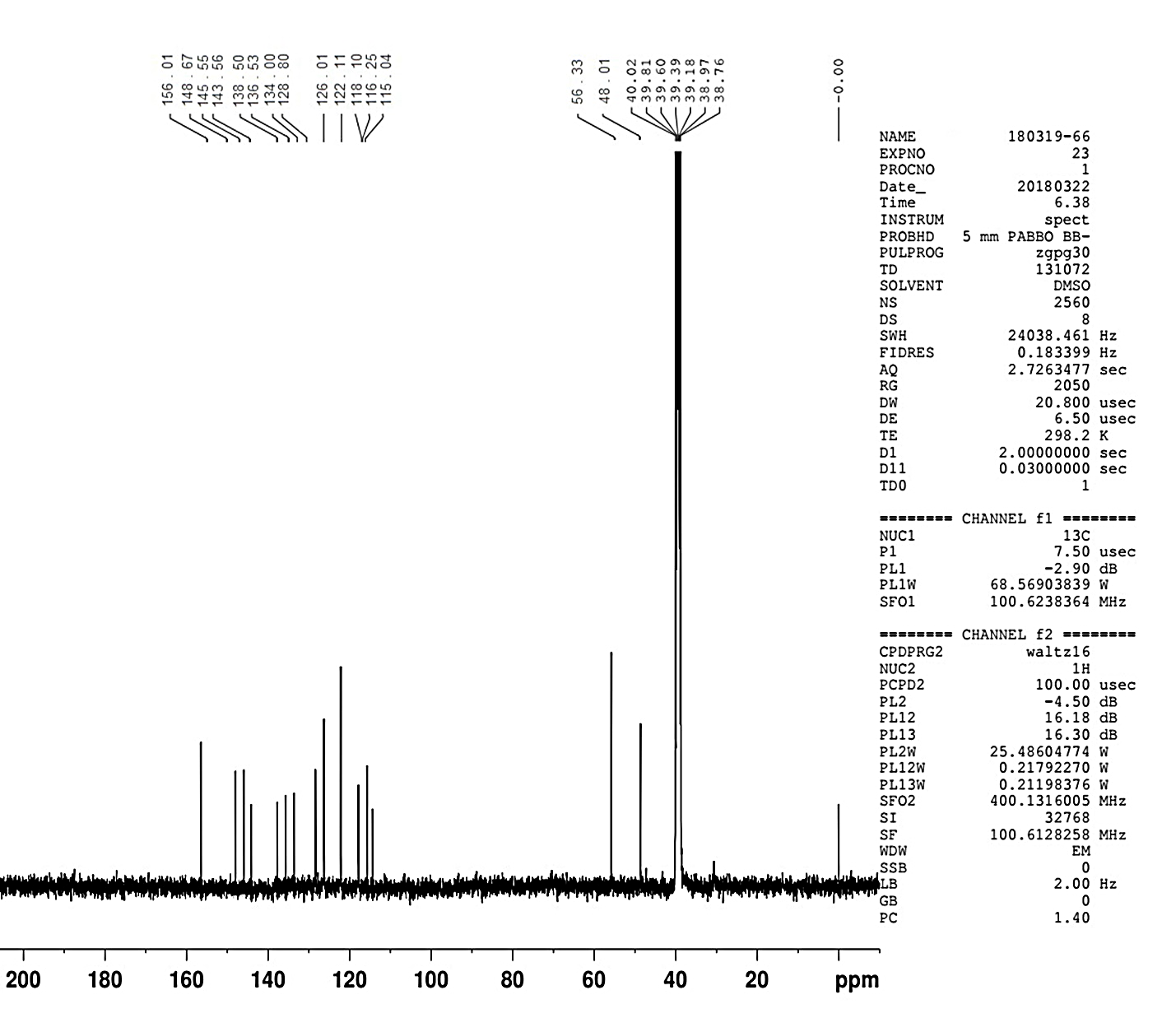


**^13^C NMR ETC-6**


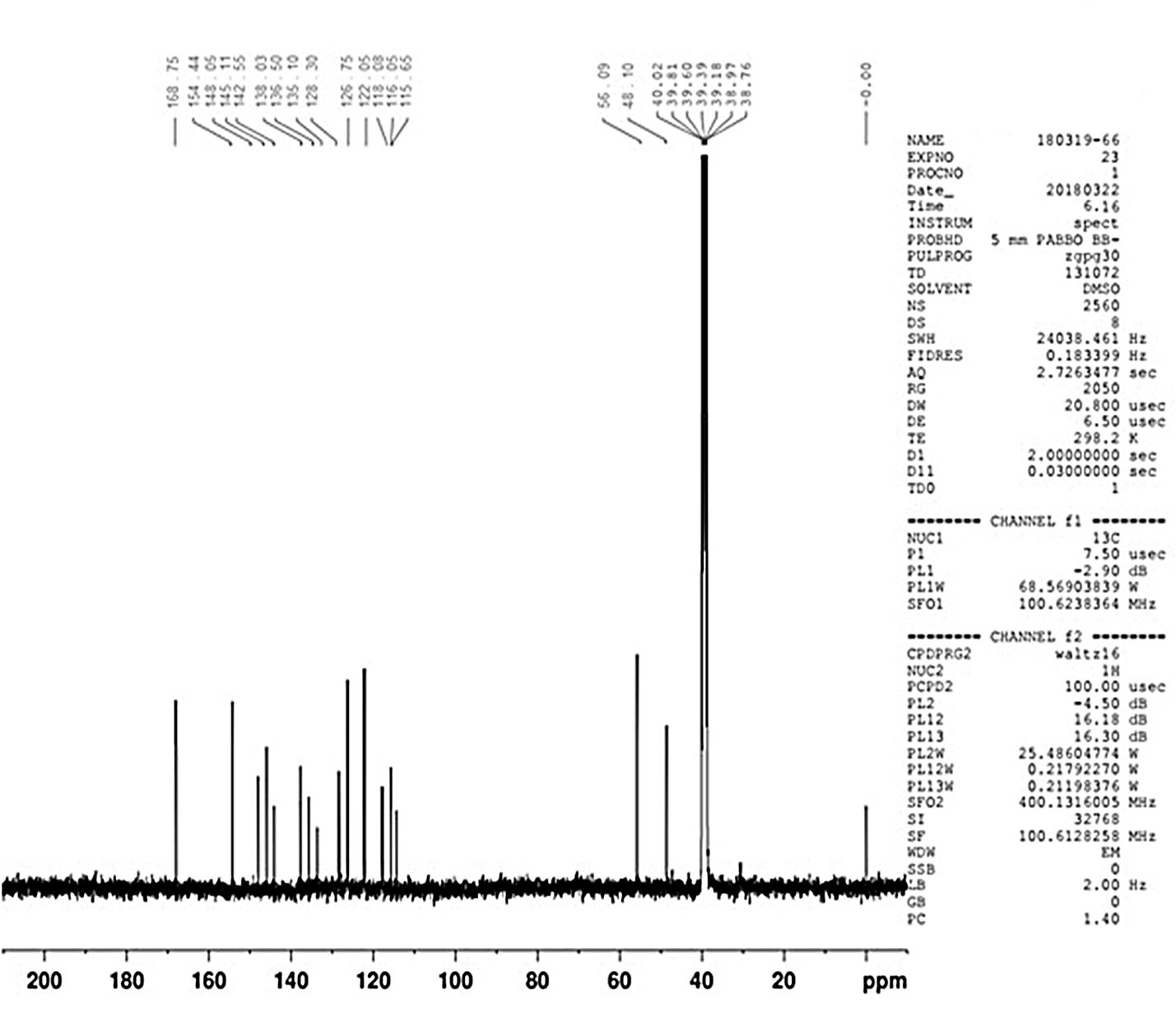


**^13^C NMR ETC-7**

**^1^HNMR Spectra ETC-1 – ETC-7**


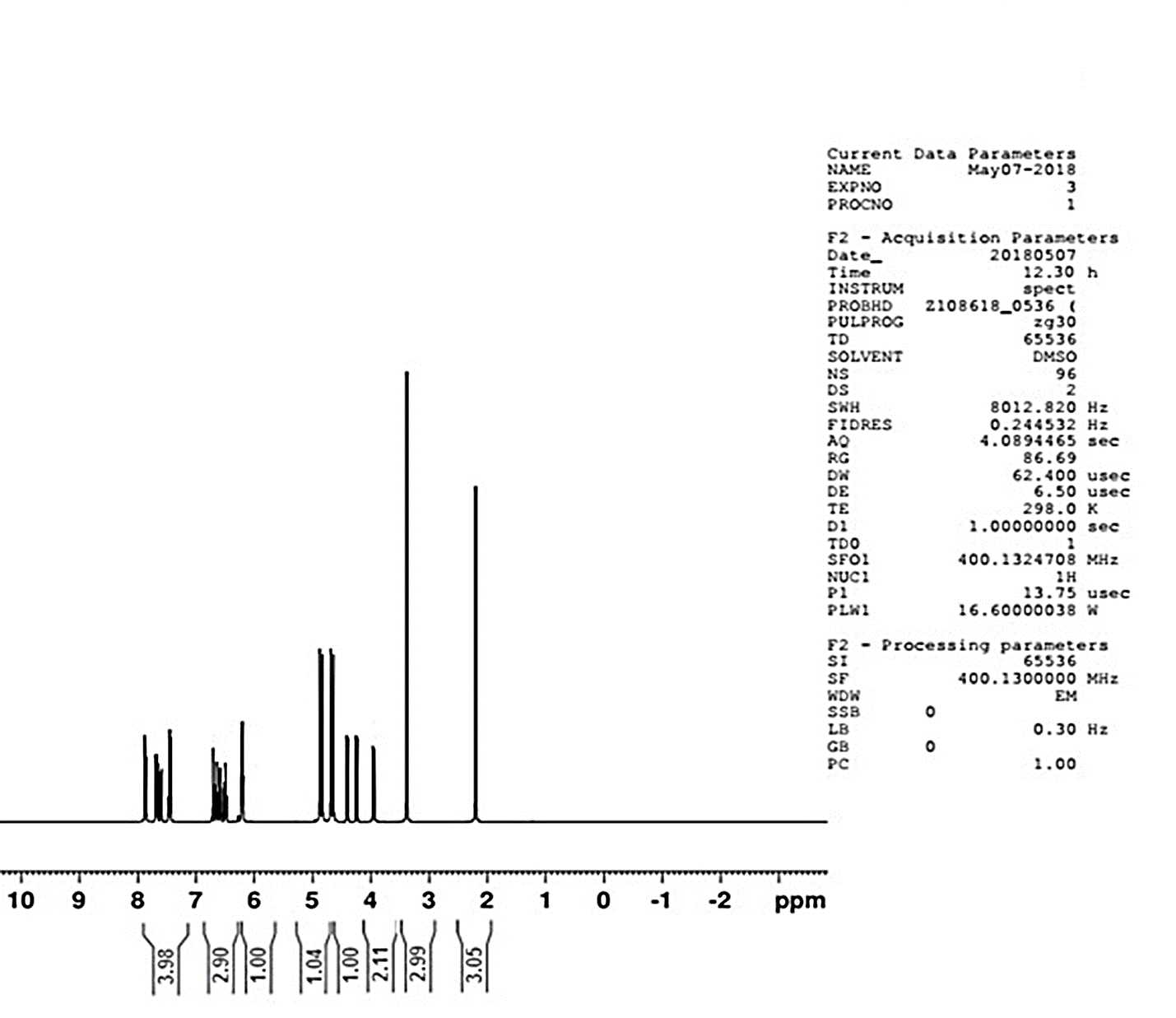


**^1^HNMR ETC-1**


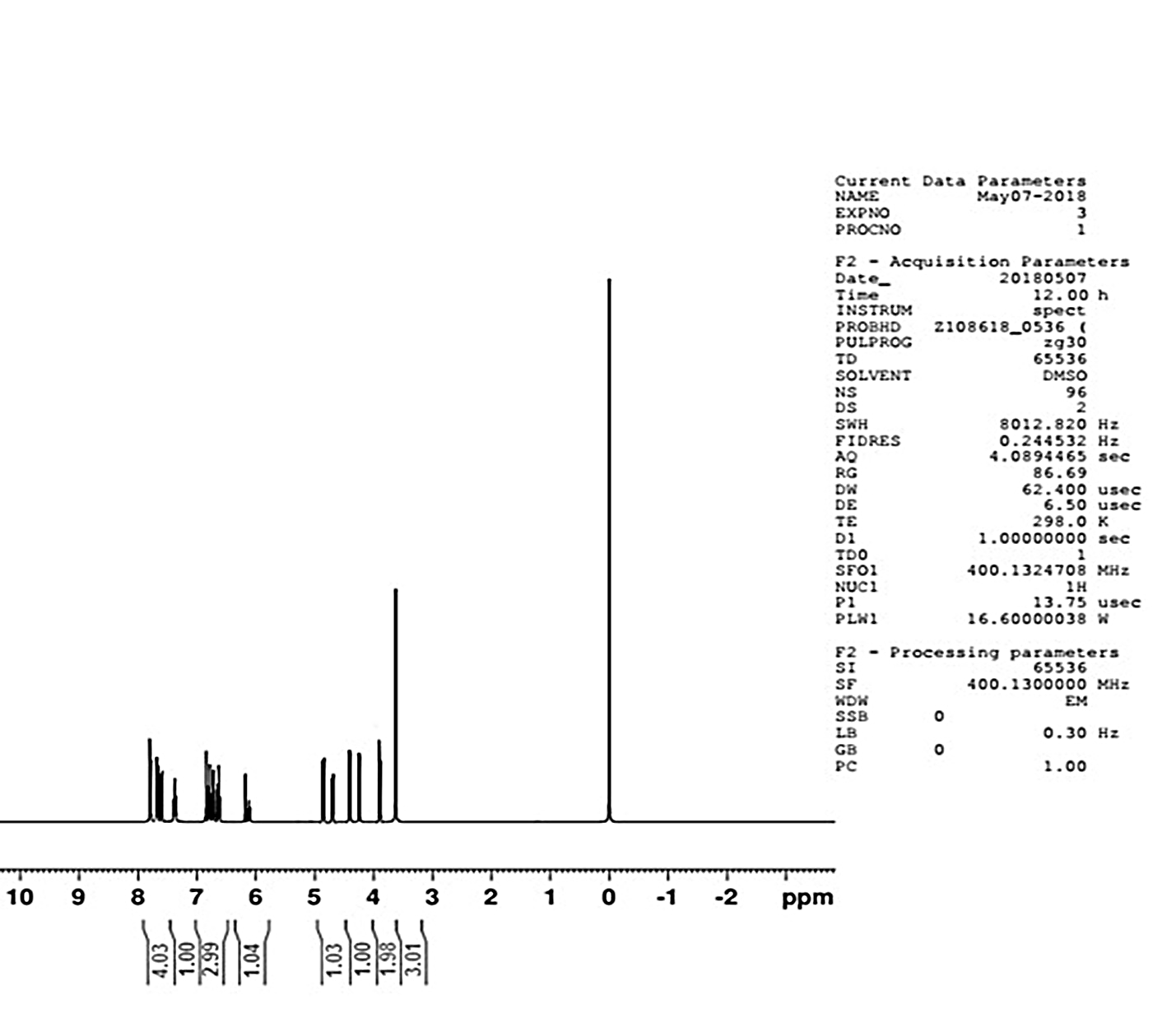


**^1^HNMR ETC-2**


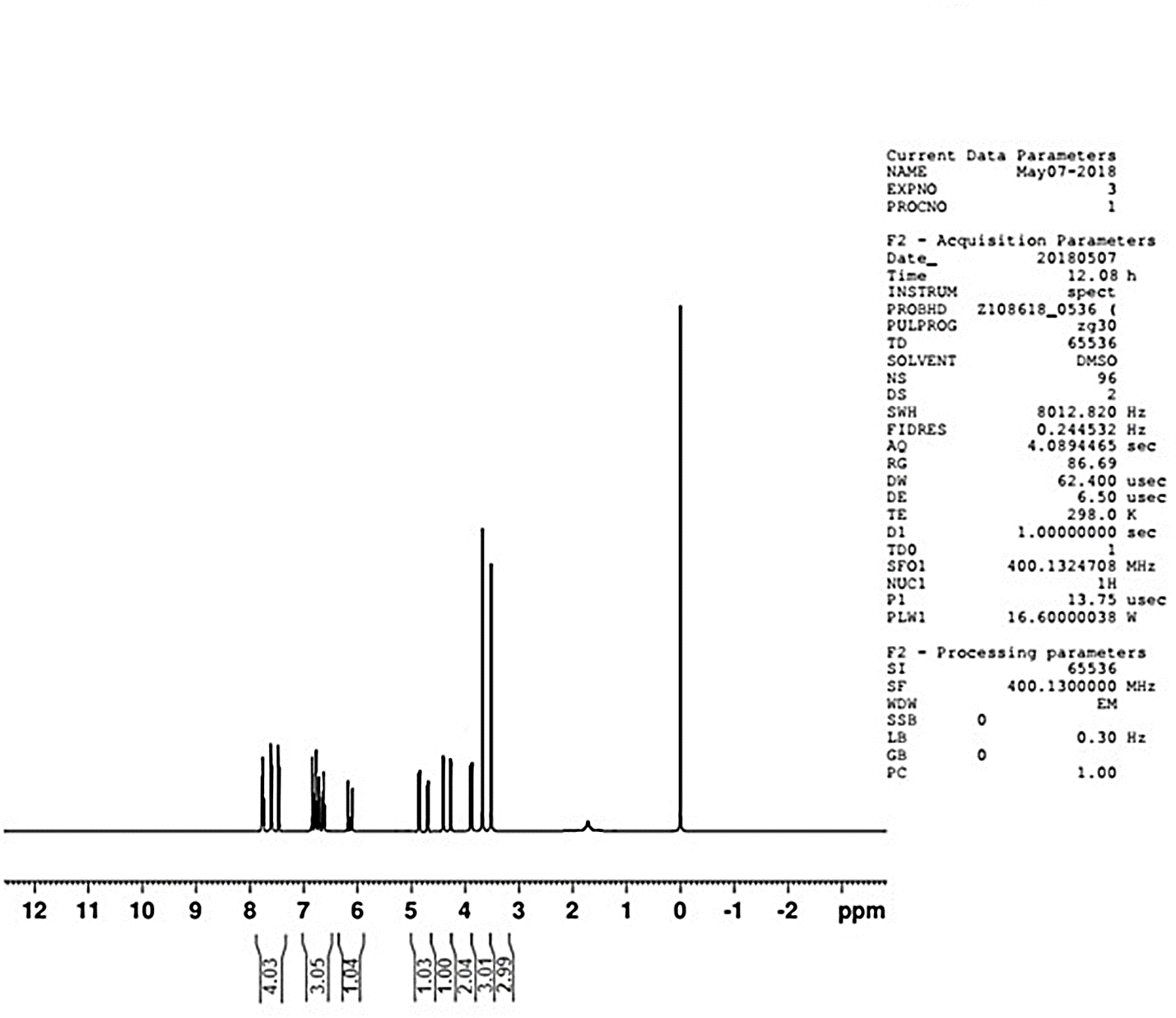


**^1^HNMR ETC-3**


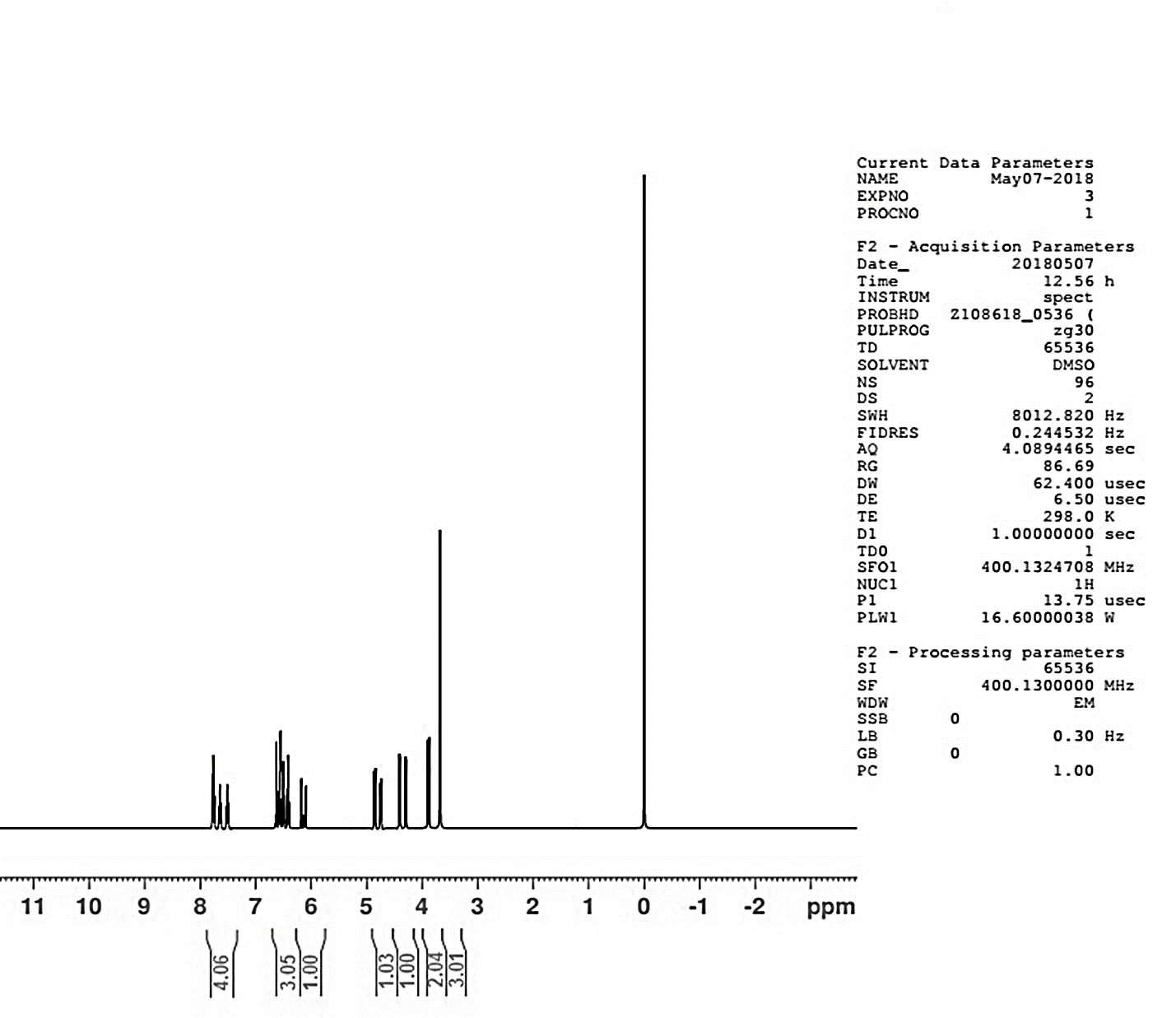


**^1^HNMR ETC-4**


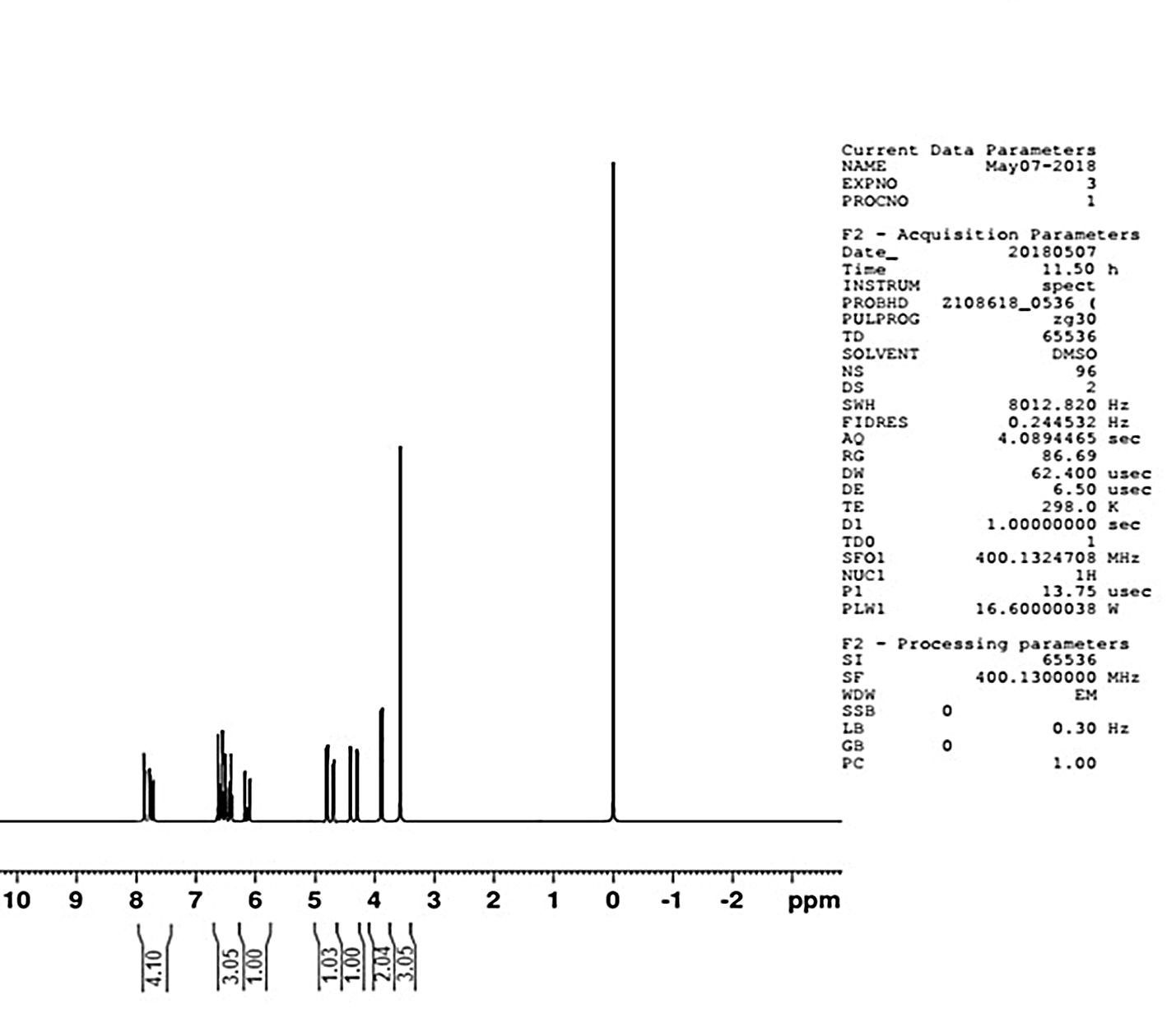


**^1^HNMR ETC-5**


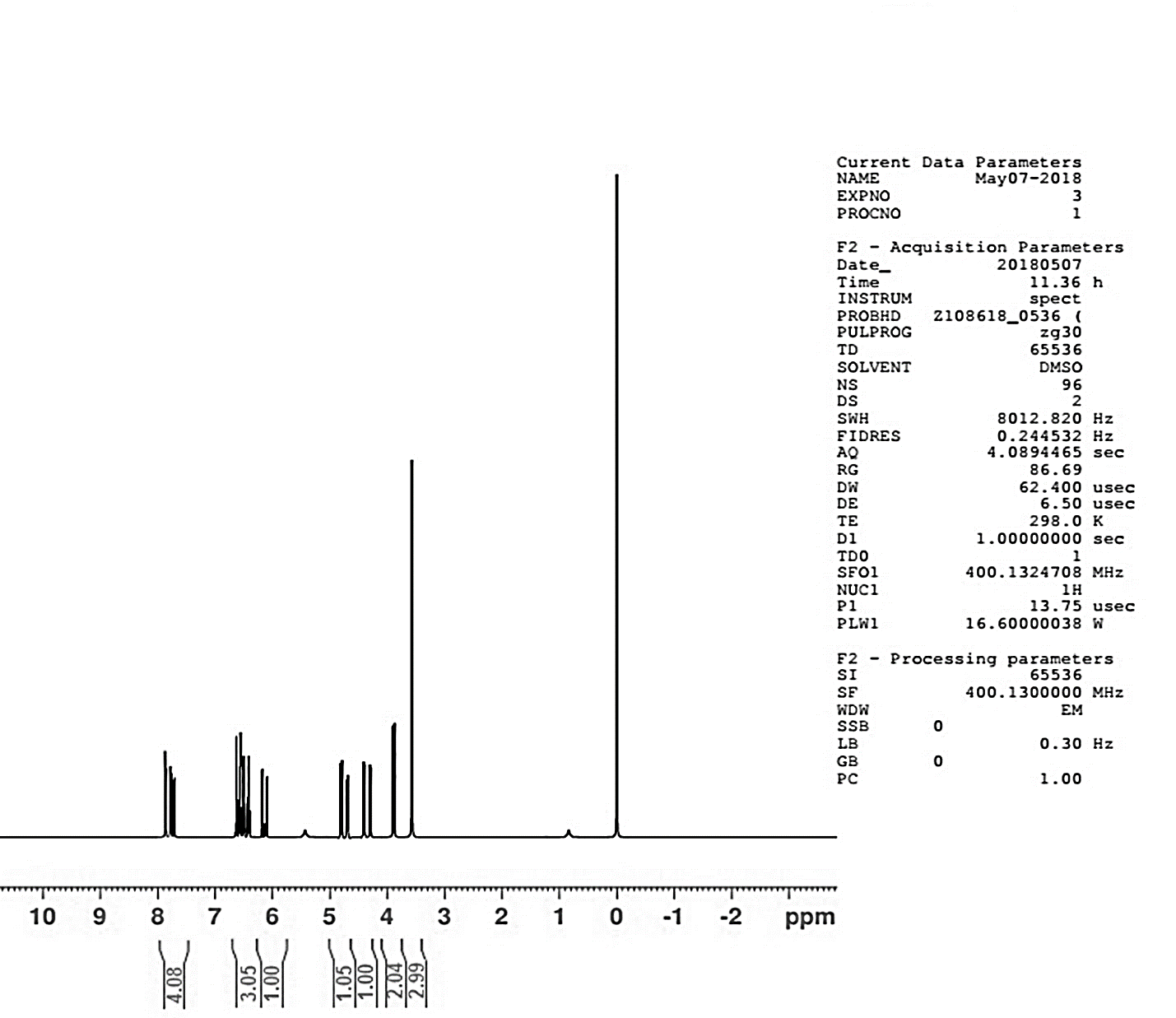


**^1^HNMR ETC-6**


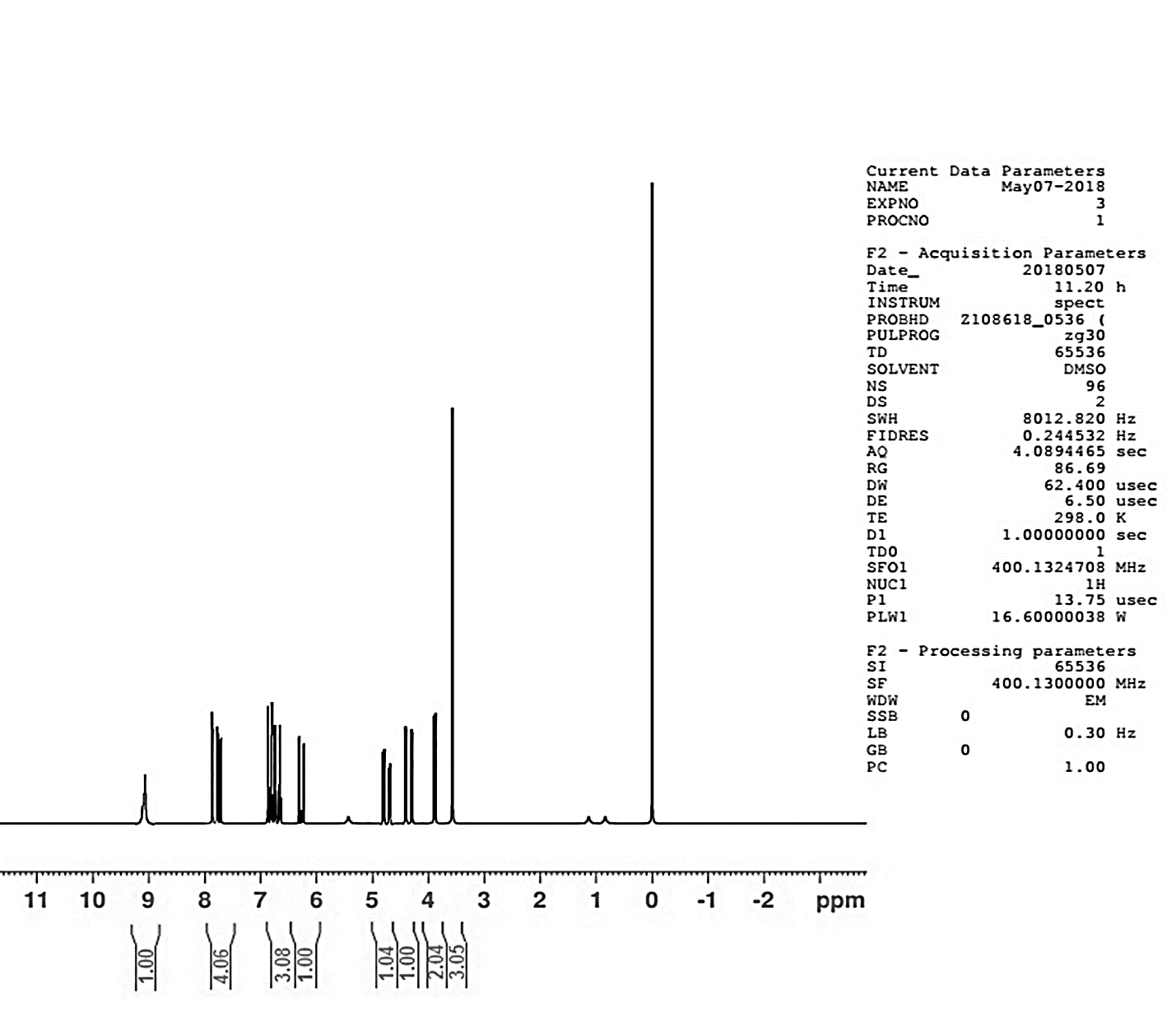


**^1^HNMR ETC-7**

**Figure S1:** ^13^C NMR and ^1^HNMR Spectra of the derivatives ETC-1 to ETC-7


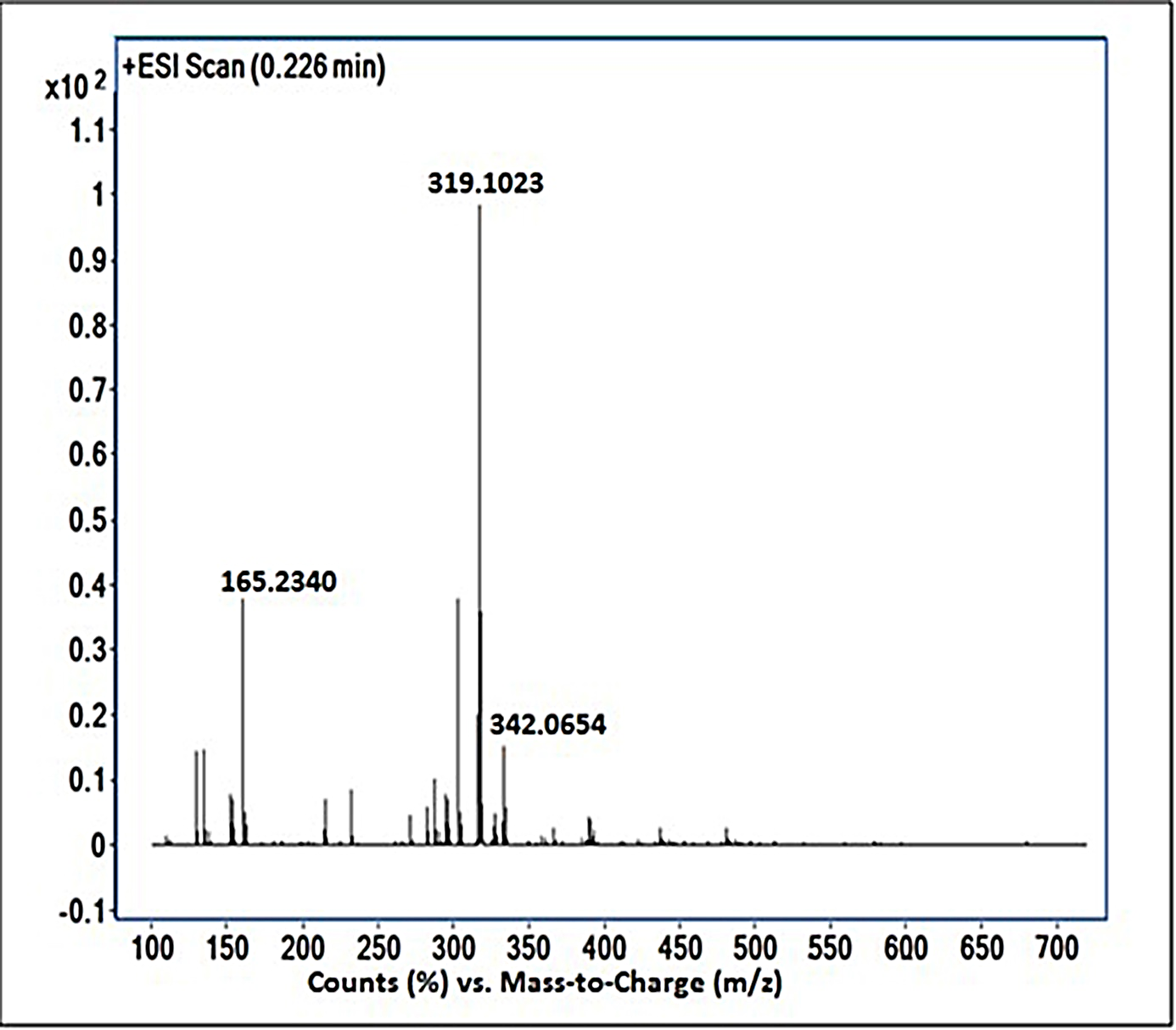


**ESI MS ETC-1**


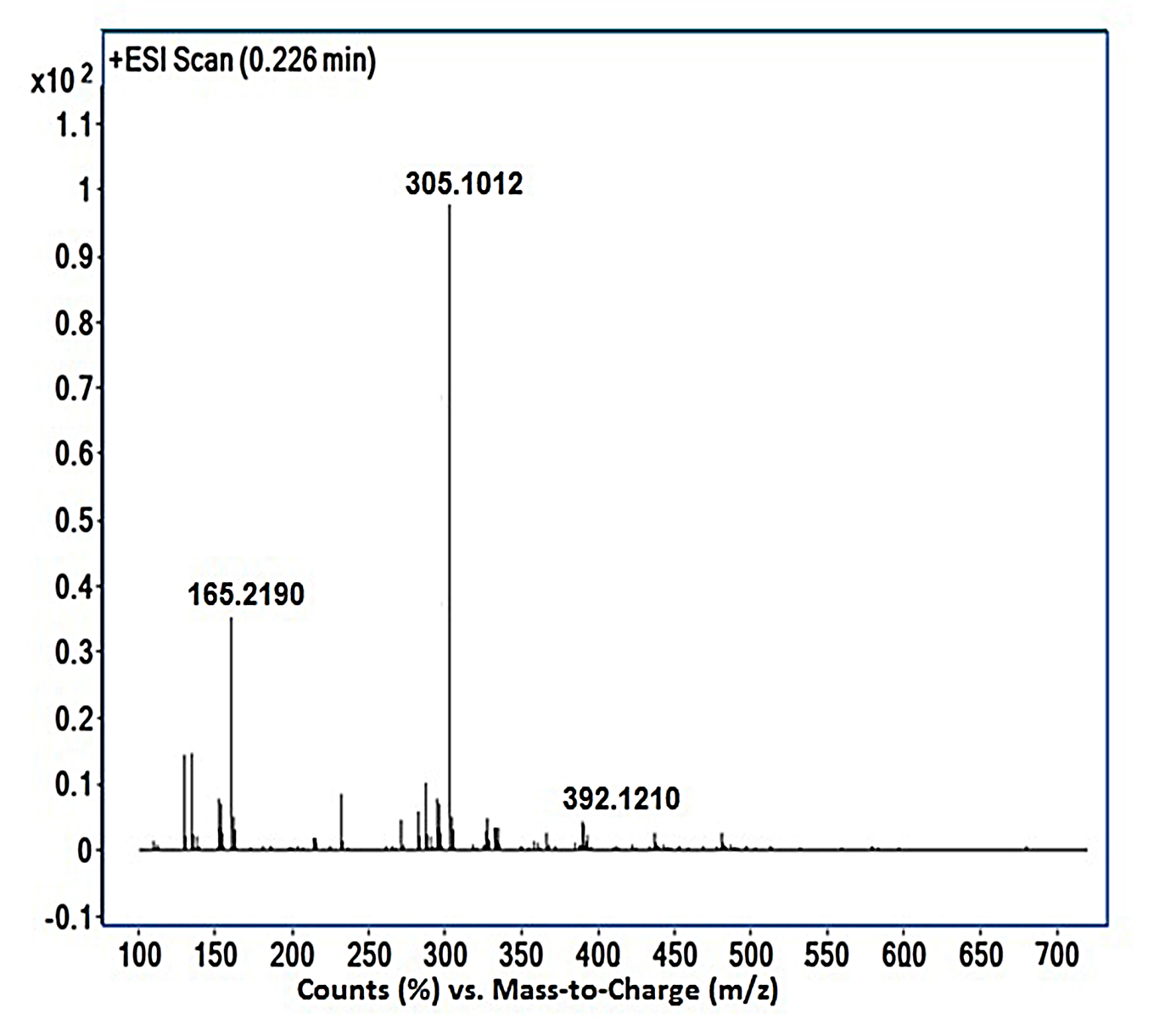


**ESI MS ETC-2**


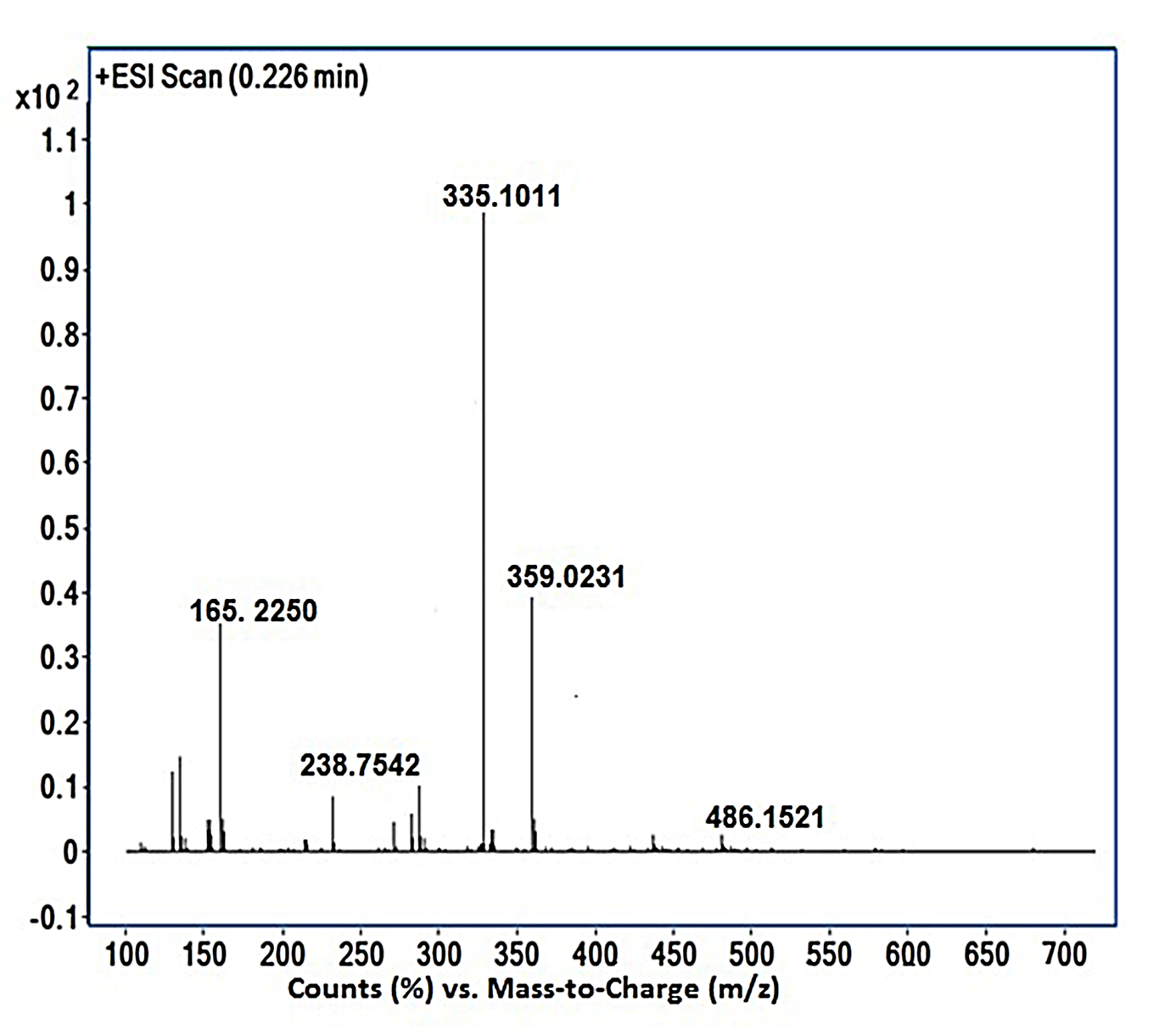


**ESI MS ETC-3**


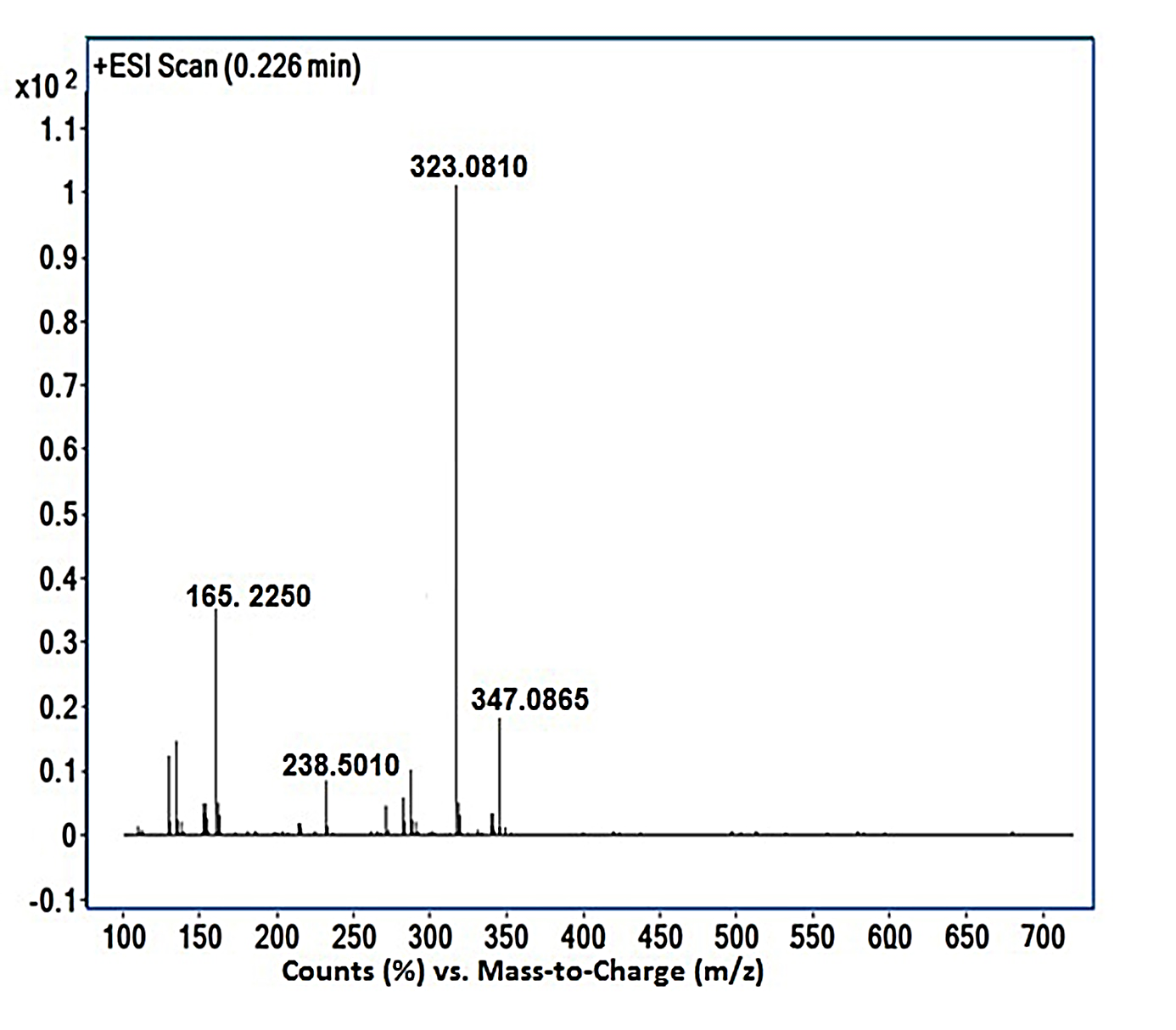


**ESI MS ETC-4**


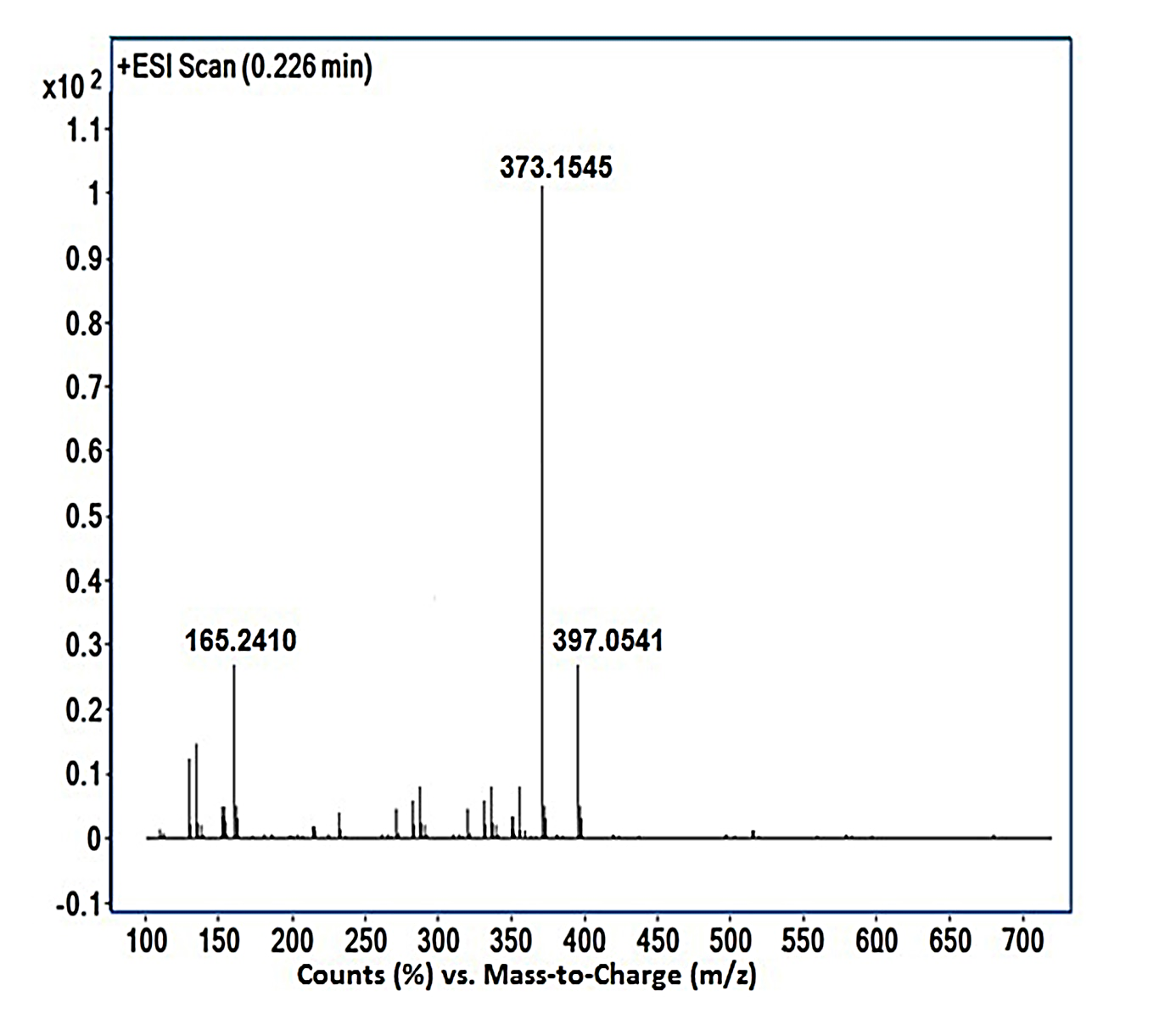


**ESI MS ETC-5**


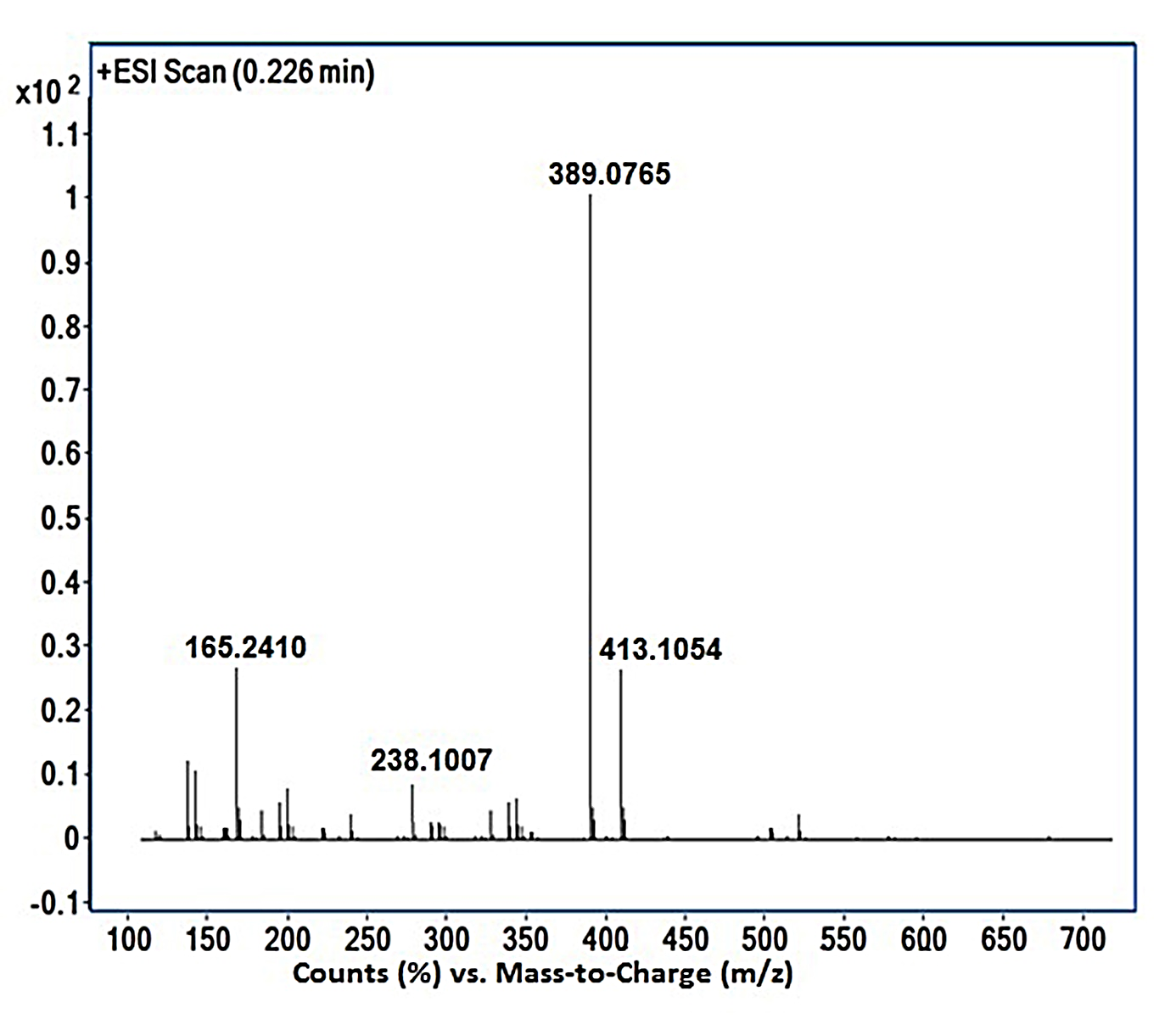


**ESI MS ETC-6**


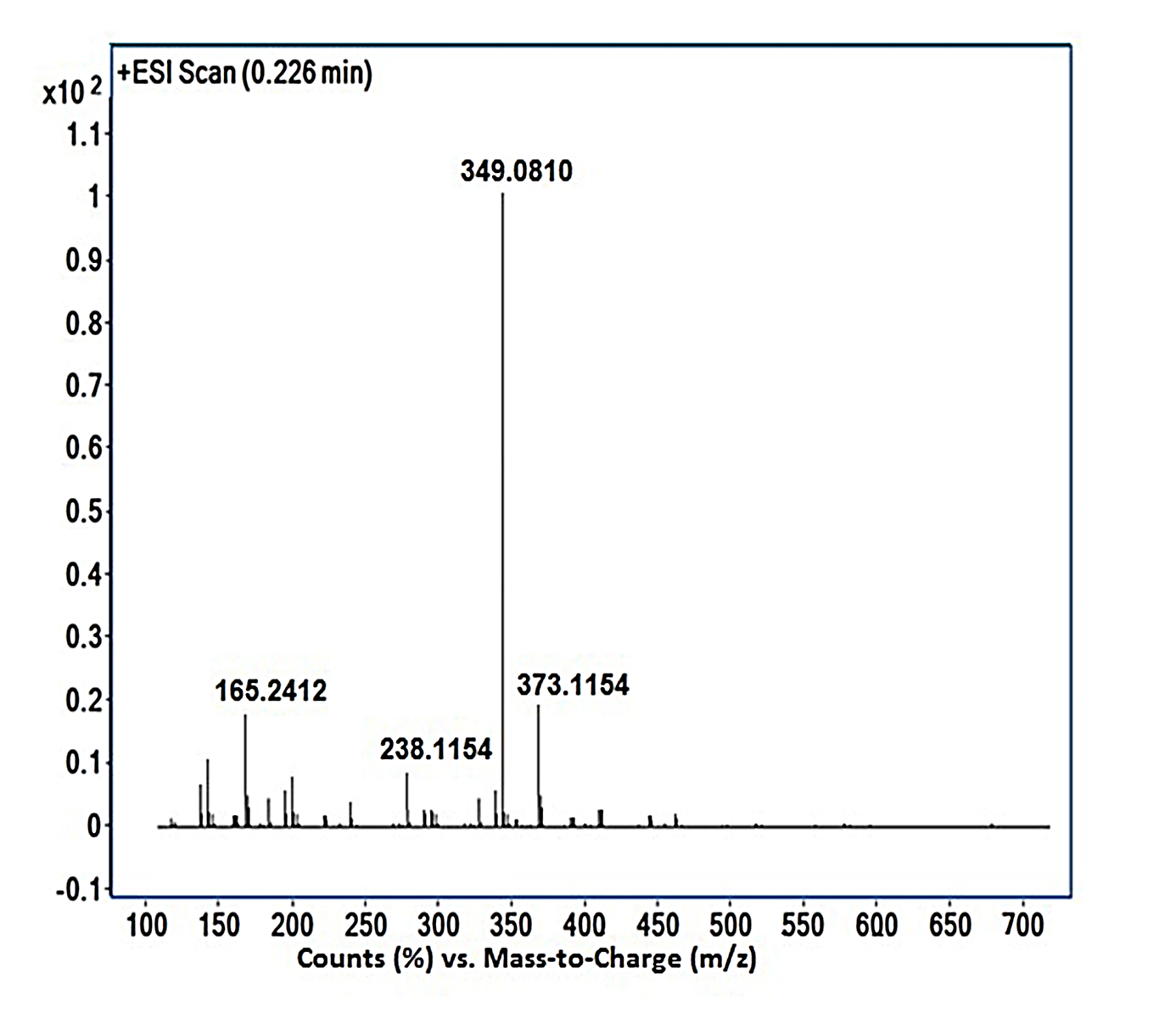


**ESI MS ETC-7**

**Figure S2: ESI MS of derivatives ETC-1 – ETC-7**

**0.5 × MIC 1 × MIC 2 × MIC**


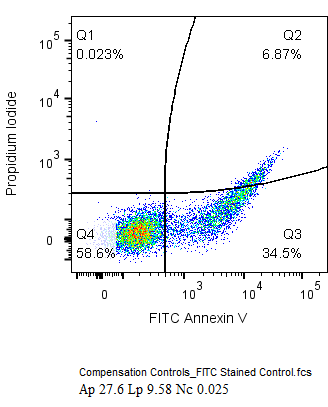

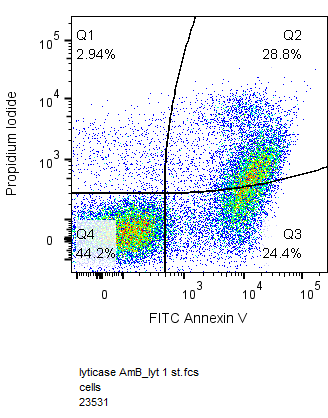

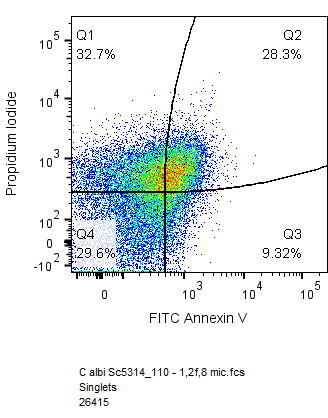
 **ETC- 5 (A)**


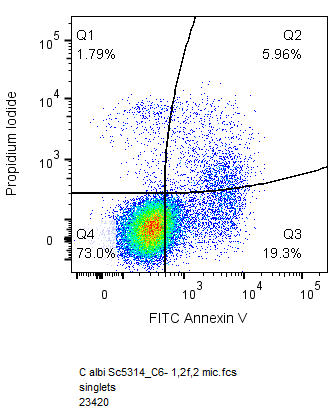

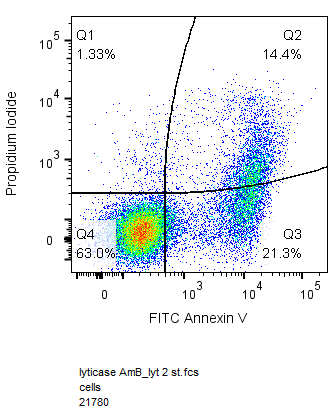

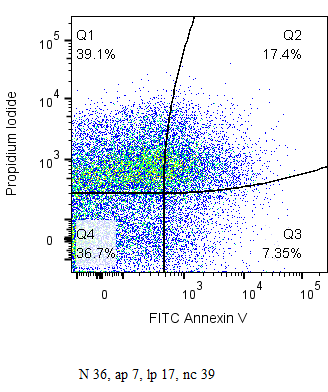
 **ETC- 6 (A)**


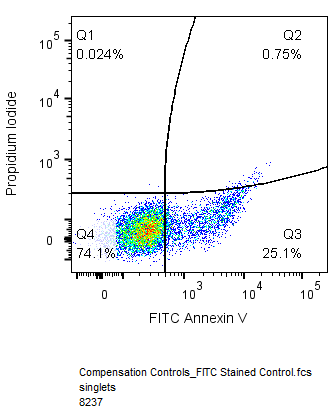

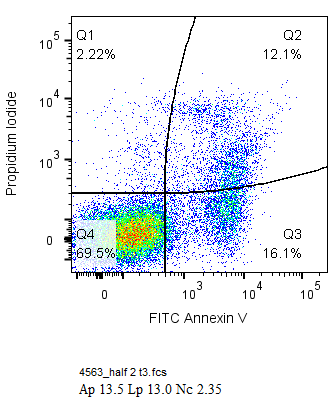

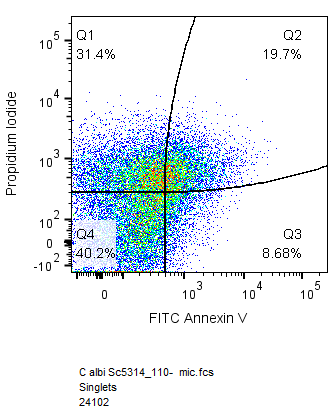
 **ETC- 7 (A)**


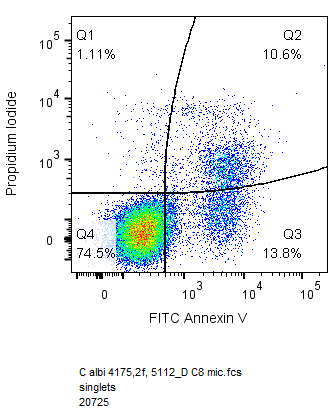

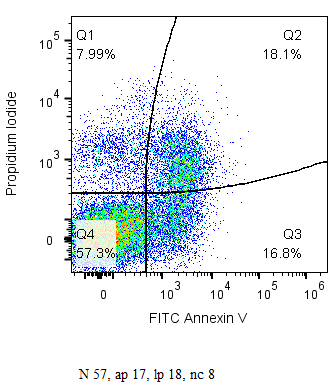

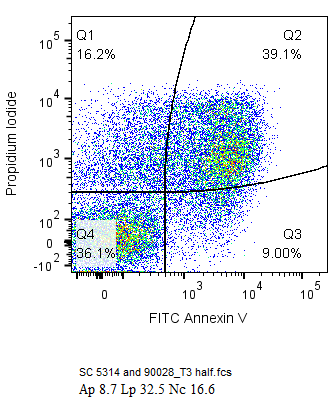
 **ETC- 5 (B)**


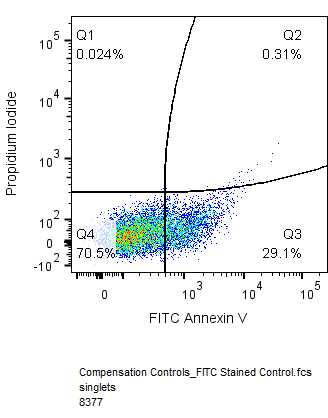

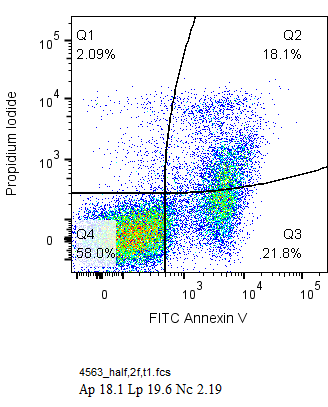

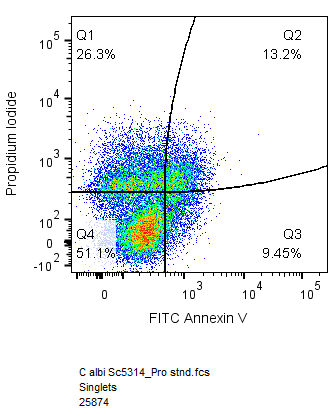
 **ETC- 6 (B)**


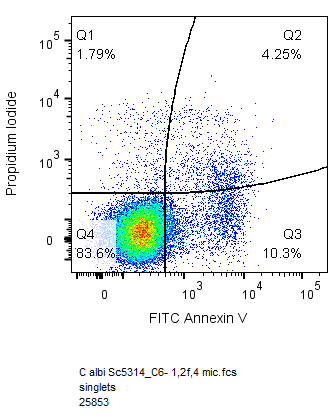

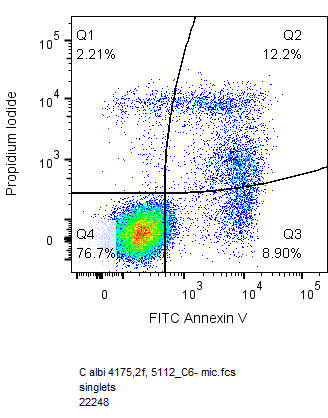

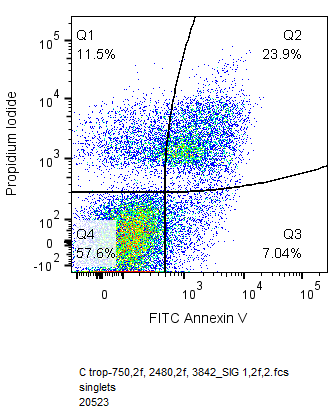
 **ETC- 7 (B)**

**Figure S3:** Cell apoptosis measured by flowcytometry using Annexin V-FITC and PI double staining. Cells were exposed to varying concentrations of test compounds (ETC-5, ETC-6 and ETC-7). In each density plot quadrant Q1: shows necrotic cells (annexin- PI+); Q2: late apoptotic cells (annexin+ PI+); Q3: early apoptotic cells (annexin+ PI-) and Q4: shows viable cells (annexin- PI-). Alphabets; represents *Candida albicans* strains; A: fluconazole susceptible *C. albicans* 4175 and B: fluconazole resistant *C. albicans* 5112.


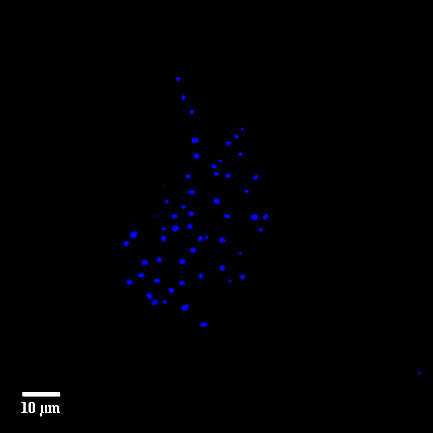

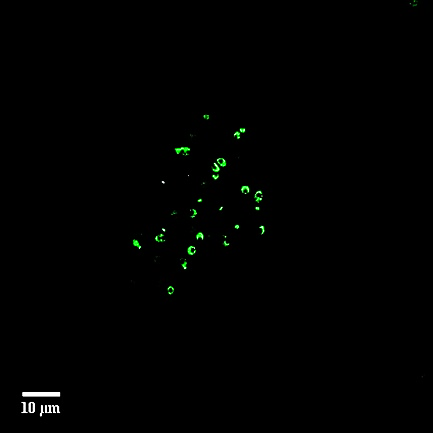

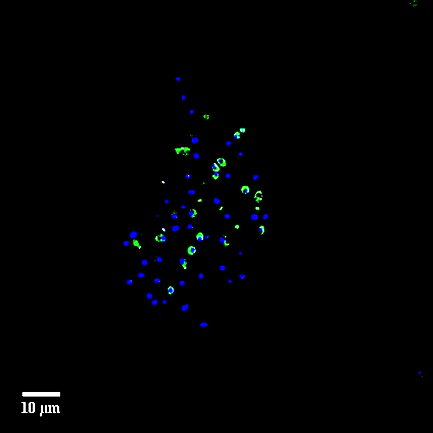
ETC-5 (0.5 × MIC)

**B3**

**B2**

**B1**


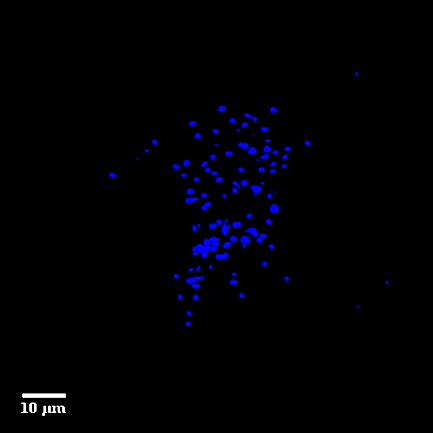

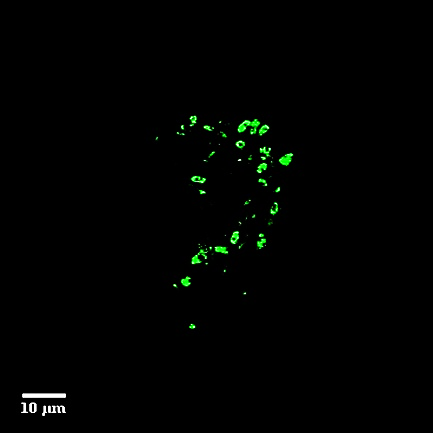

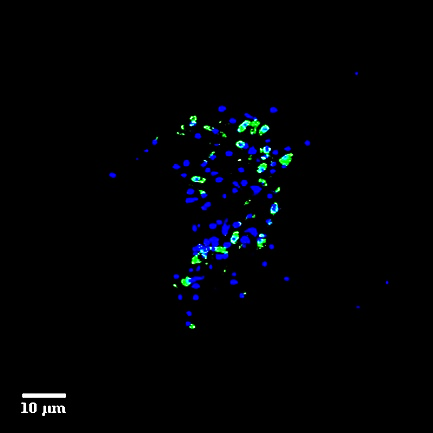
ETC-5 (1 × MIC)

**B3**

**B2**

**B1**


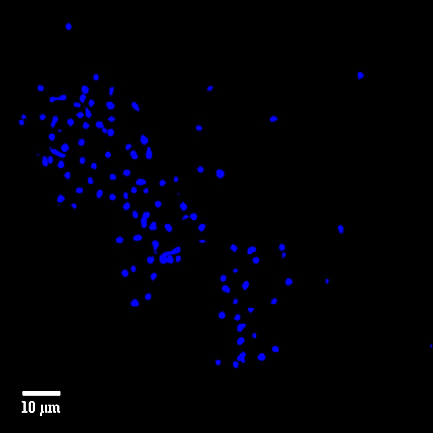

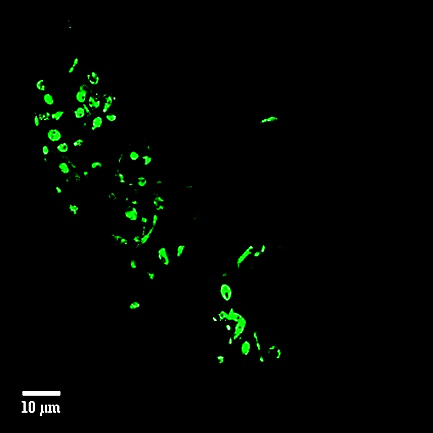

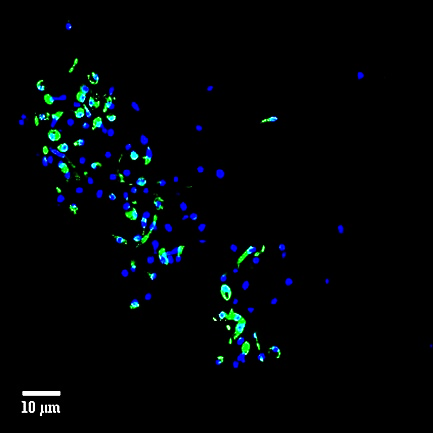
ETC-5 (2 × MIC)

**B3**

**B2**

**B1**


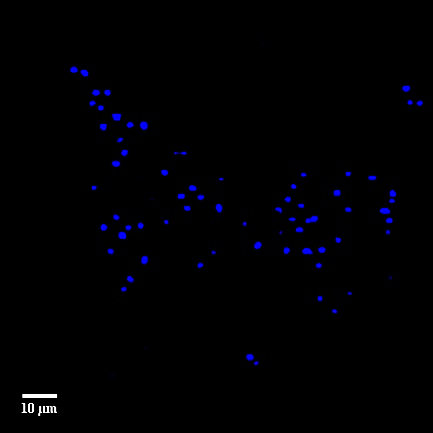

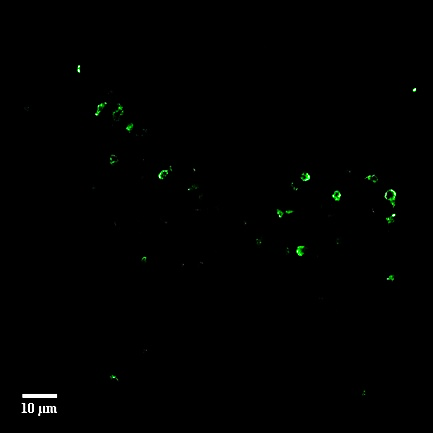

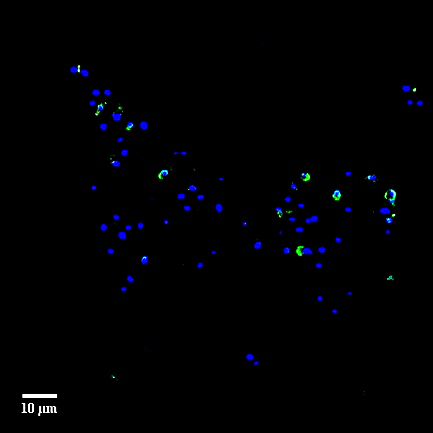
ETC-6 (0.5 × MIC)

**B3**

**B2**

**B1**


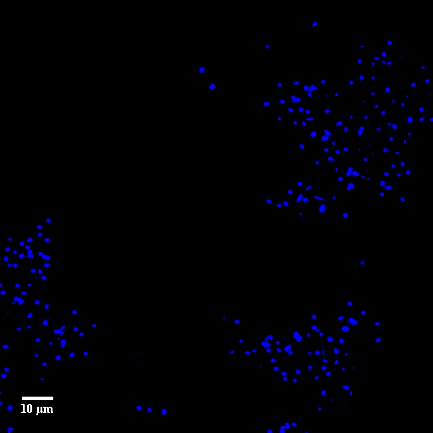

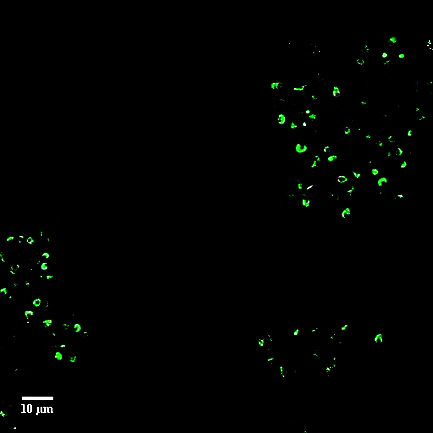

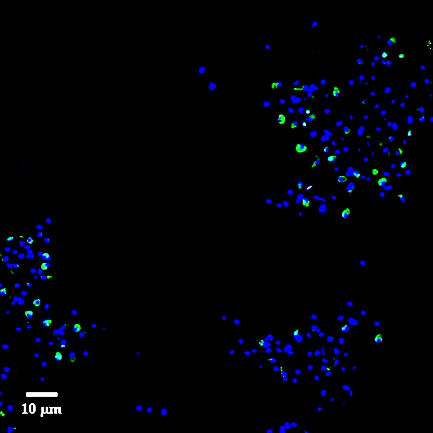
ETC-6 (1 × MIC)

**B3**

**B2**

**B1**


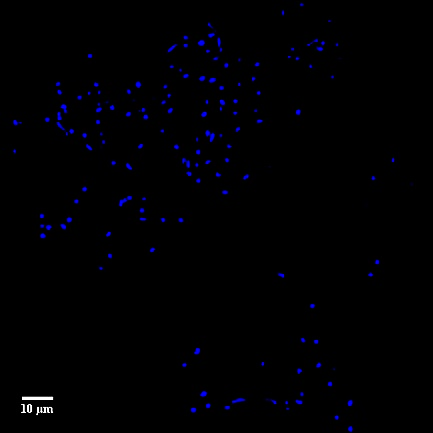

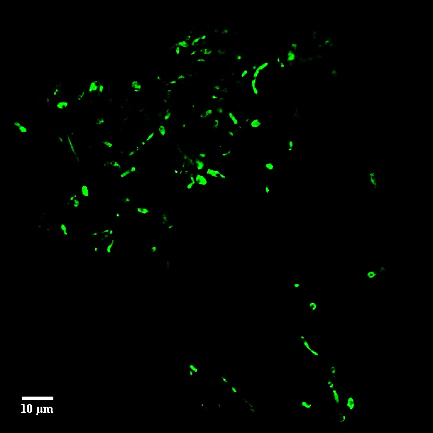

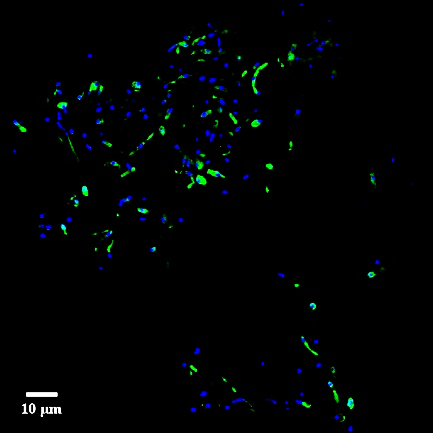
ETC-6 (2 × MIC)

**B3**

**B2**

**B1**


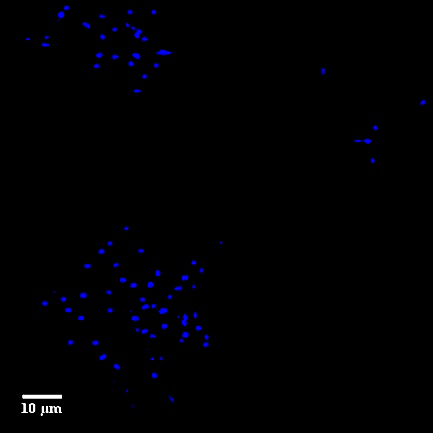

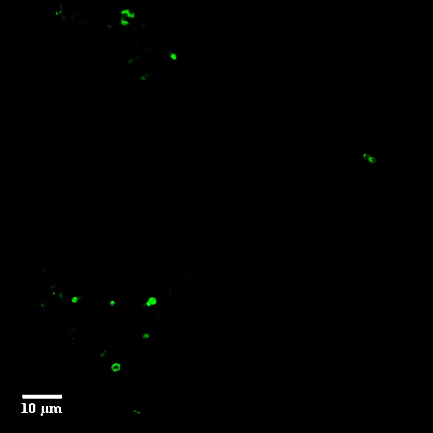

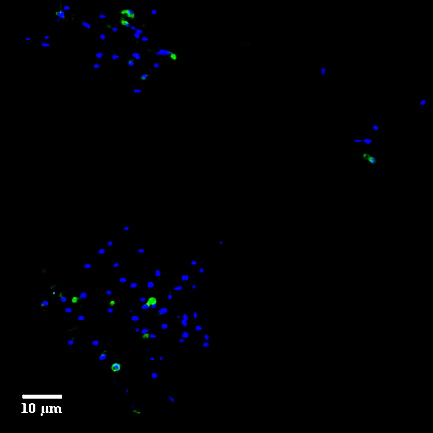
ETC-7 (0.5 × MIC)

**B3**

**B2**

**B1**


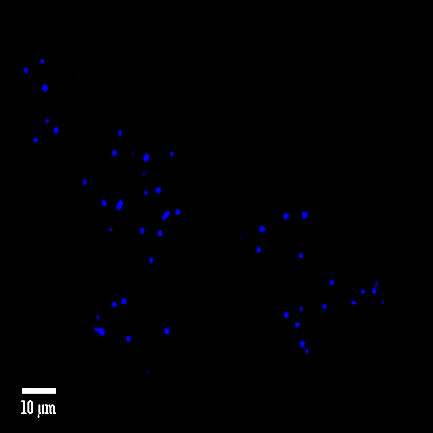

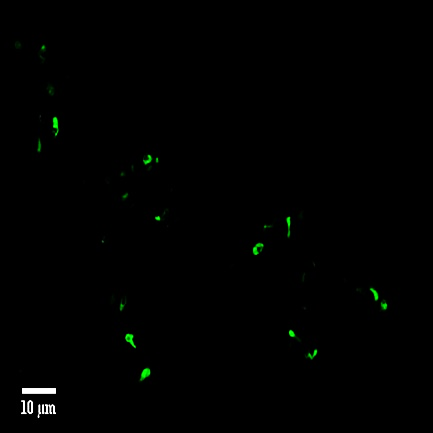

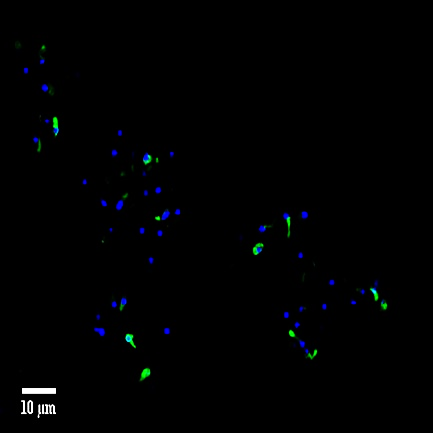
ETC-7 (1 × MIC)

**B3**

**B2**

**B1**


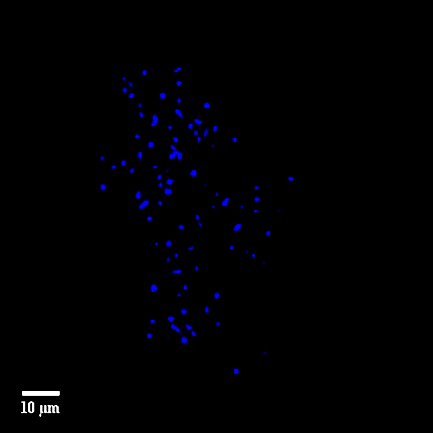

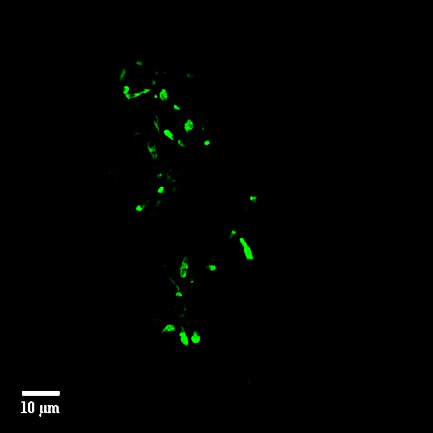

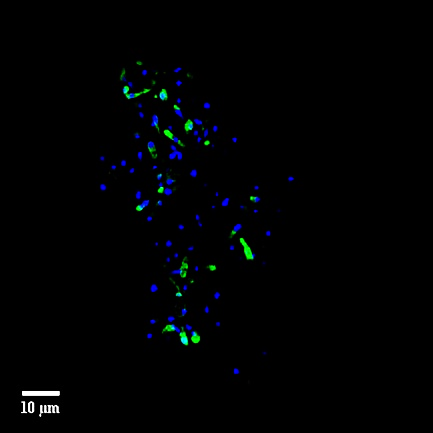
ETC-7 (2 × MIC)

**B1**

**B3**

**B2**


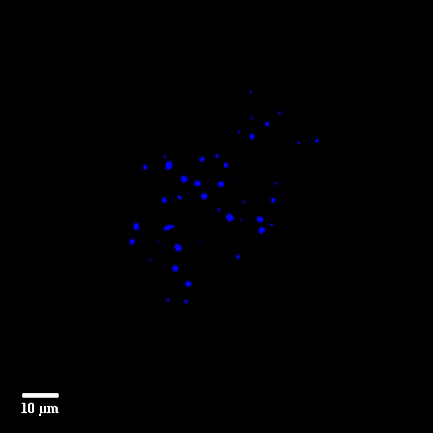

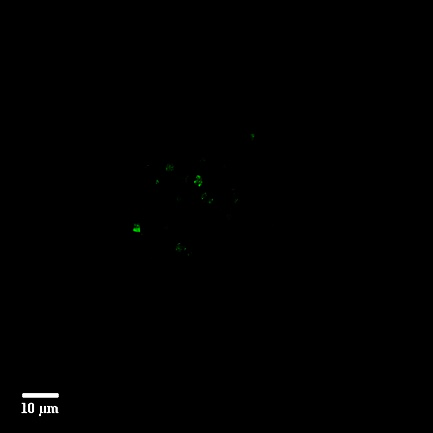

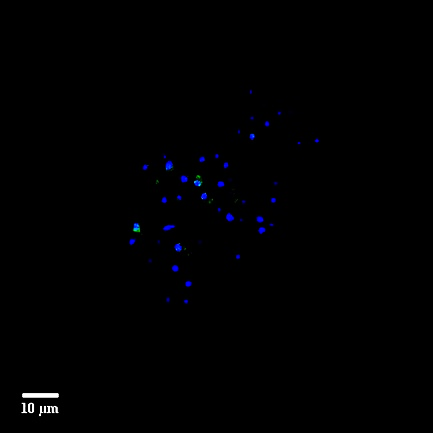
ETC-5 (0.5 × MIC)

**C2**

**C1**

**C3**


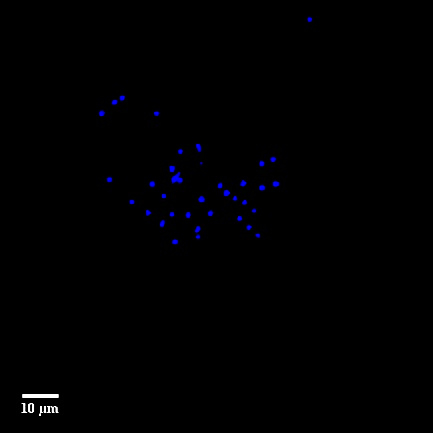

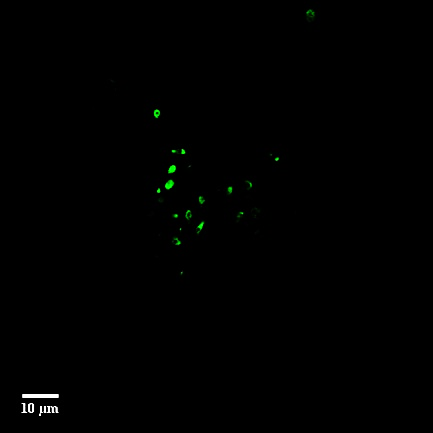

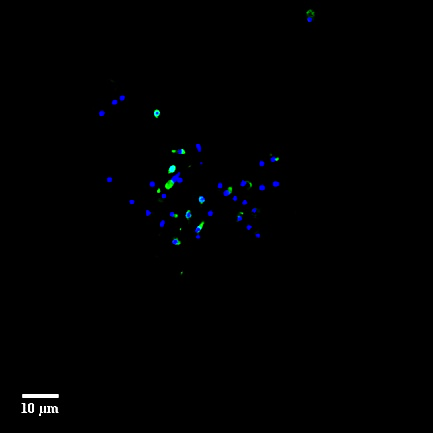
ETC-5 (1 × MIC)

**C3**

**C2**

**C1**


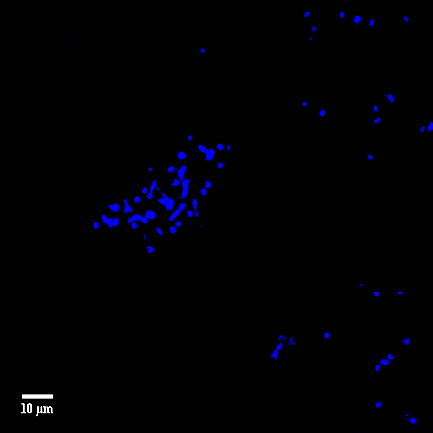

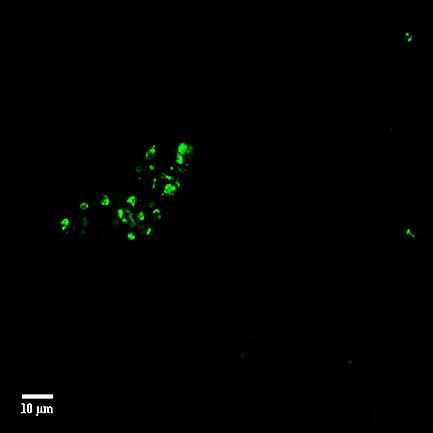

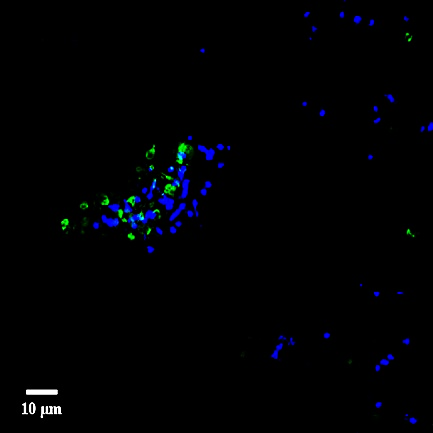
ETC-5 (2 × MIC)

**C3**

**C2**

**C1**


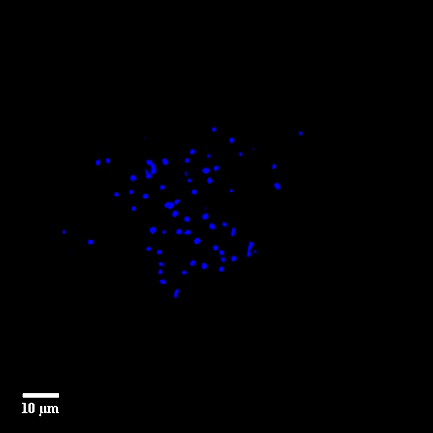

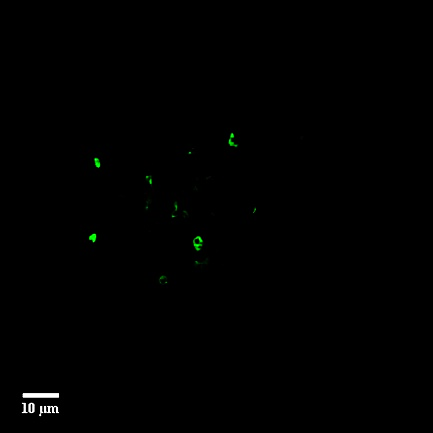

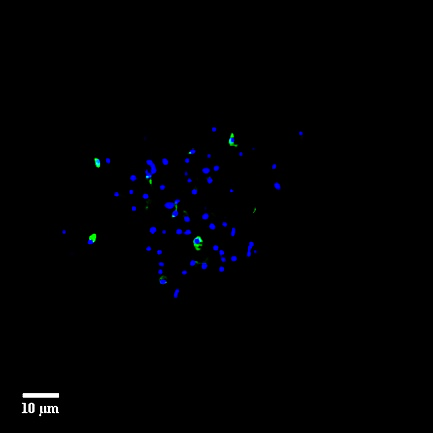
ETC-6 (0.5 × MIC)

**C2**

**C1**

**C3**


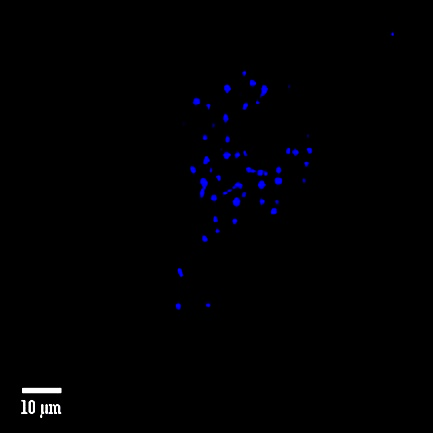

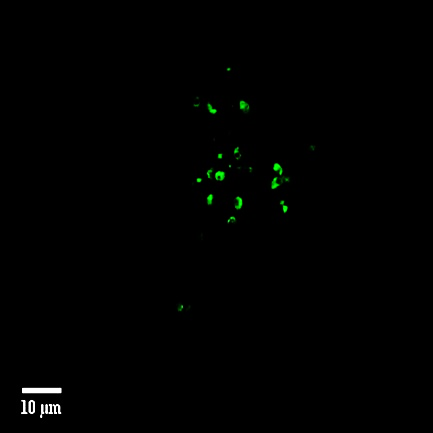

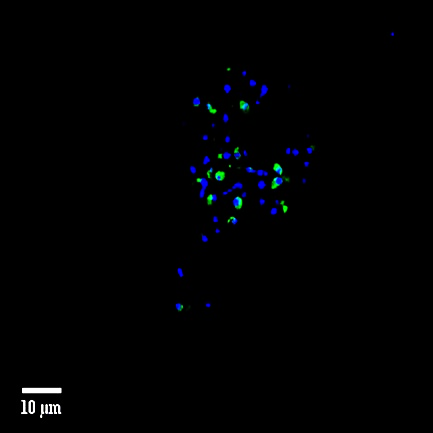
ETC-6 (1 × MIC)

**C2**

**C1**

**C3**


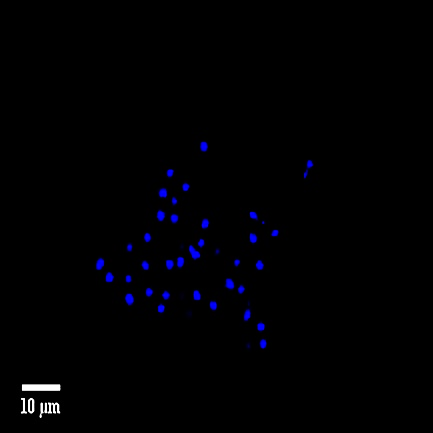

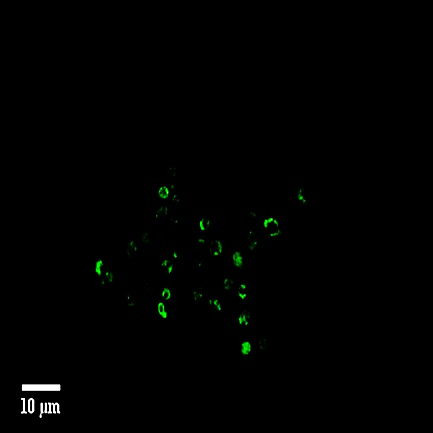

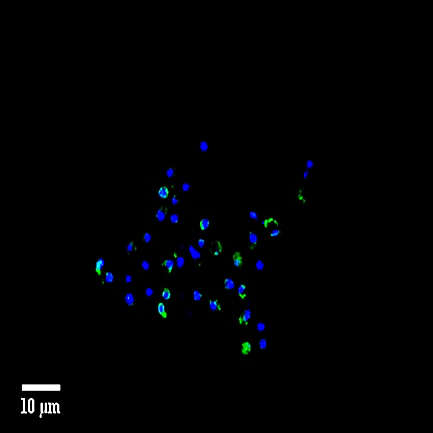
ETC-6 (2 × MIC)

**C1**

**C3**

**C2**


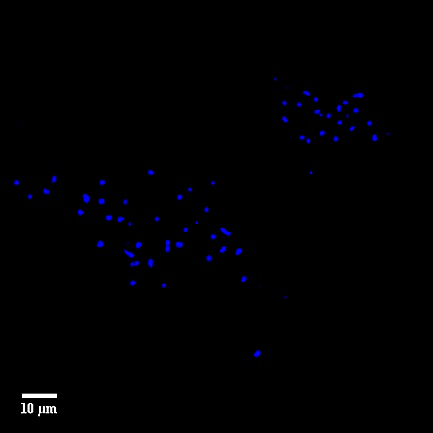

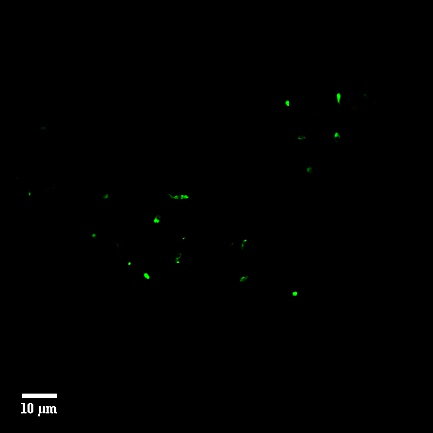

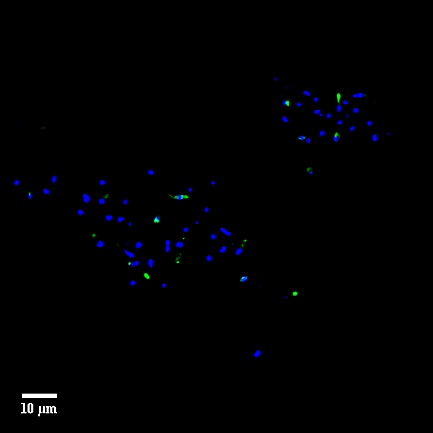
ETC-7 (0.5 × MIC)

**C2**

**C3**

**C1**


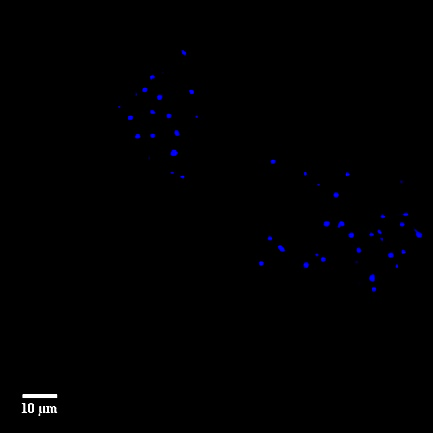

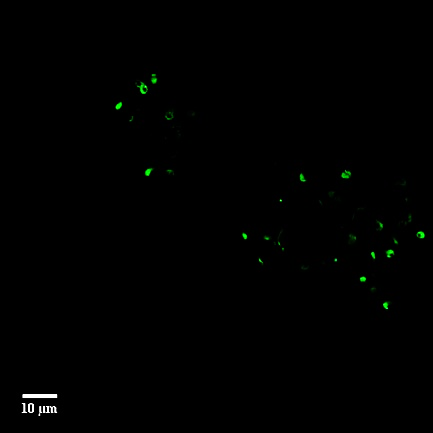

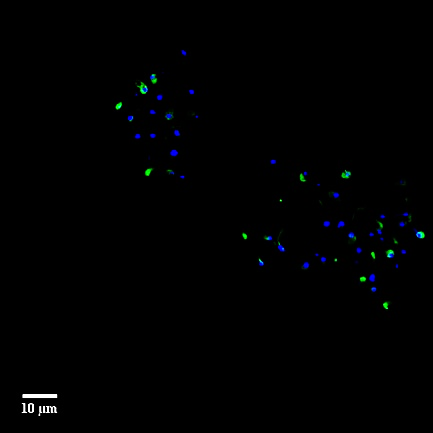
ETC-7 (1 × MIC)

**C1**

**C2**

**C3**


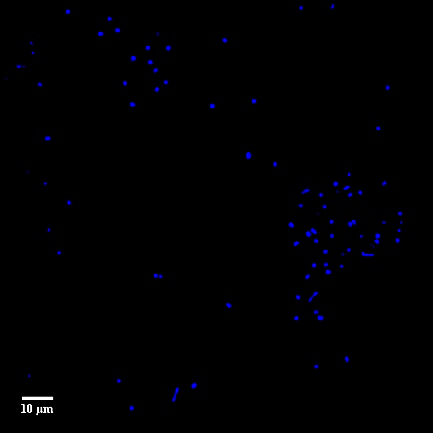

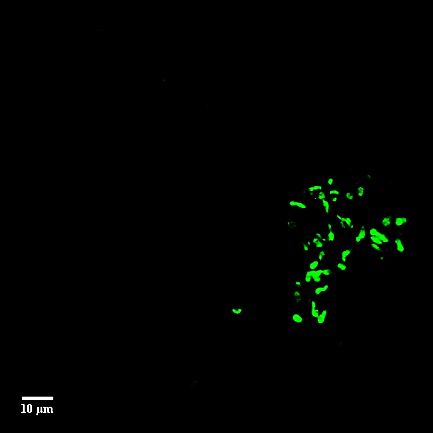

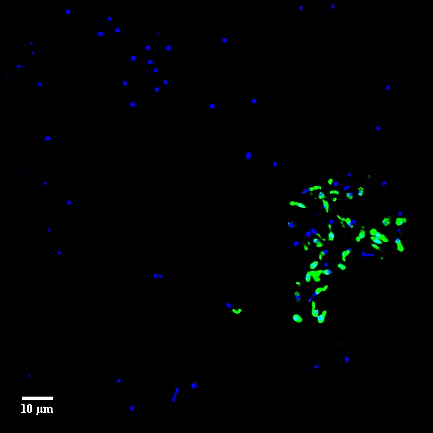
ETC-7 (2 × MIC)

**C2**

**C1**

**C3**

**Figure S4:** Confocal scanning fluorescence images of *C. albicans* 4175 (B) and *C. albicans* 5112 (C) cells after exposed to varying concentrations of ETC-5, ETC-6 and ETC-7. 1, 2 and 3 represents live or dead intact cells stained with Hoechst 33342 dye (blue fluorescence), apoptotic cells indicating active DNA fragmentation stained with Alexa Fluor 488 (green fluorescence) and merged image respectively. Scale bar: 10 µm.
